# Sorcin couples Annexin A11 recruitment to ESCRT-III assembly for plasma membrane repair

**DOI:** 10.1073/pnas.2610420123

**Published:** 2026-08-10

**Authors:** Jordan Matthew Ngo, Justin Krish Williams, Abinayaa Murugupandiyan, Randy Schekman

**Affiliations:** ^a^ Department of Molecular and Cell Biology, University of California, Berkeley, CA 94720; ^b^ HHMI, University of California, Berkeley, CA 94720

**Keywords:** plasma membrane, membrane repair, endosomal sorting complex required for transport (ESCRT), annexin

## Abstract

The absence of a cell wall affords animal cells diverse functionality at the cost of acute sensitization to plasma membrane (PM) damage. Thus, animal cells tightly monitor and maintain PM integrity to prevent cell death. Genetic loss of PM repair factors is associated with human diseases such as muscular dystrophy. Despite evidence that annexin and endosomal sorting complex required for transport (ESCRT) proteins are required for PM repair, the extent to which their recruitment is coordinated at sites of membrane damage remains unclear. Here, leveraging quantitative organellar proteomics and genome-wide CRISPR interference screens, we identify sorcin as a PM repair factor that couples annexin A11 (ANXA11)-mediated sensing of PM damage to ESCRT-III assembly. We show that sorcin directly binds ANXA11 and ALIX in the presence of Ca^2+^ via its penta-EF-hand domain and flexible N terminus, respectively, and is required for ESCRT-III recruitment to PM lesions and membrane resealing. Our data support a model in which ANXA11, recruited to the PM upon damage-induced Ca^2+^ influx, serves as an anchor that facilitates the sequential recruitment of sorcin and ESCRT-III at PM lesions. Together, these findings establish a Ca^2+^-dependent scaffolding mechanism that couples PM damage sensing to ESCRT-III assembly for PM repair.

## Significance

The lack of a cell wall affords animal cells diverse functionality but renders them vulnerable to plasma membrane (PM) damage. Thus, animal cells maintain PM integrity to prevent cell death, and mutations that compromise PM repair are associated with muscular dystrophy. Despite the importance of PM repair in normal health and human disease, the mechanisms that couple PM damage sensing to repair machinery recruitment remain incompletely understood. Leveraging quantitative organellar proteomics and genome-wide CRISPR interference screens, we report the identification of sorcin as a repair factor that facilitates endosomal sorting complex required for transport (ESCRT)-III assembly at sites of PM damage marked by ANXA11. Thus, our study identifies a mechanism that couples PM damage sensing to membrane repair.

A defining feature of animal cells is the lack of a cell wall. Although this absence confers animal cells with distinct functional advantages compared to cells from other taxa, it also renders animal cells susceptible to plasma membrane (PM) damage. PM disruption is common, and a subset of cells, particularly those found in mechanically active tissues, are especially vulnerable to damage (1). For example, muscle and endothelial cells experience high levels of PM damage due to repeated contractions and fluid shear stress, respectively (2–4). If left unresolved, persistent PM damage leads to cell death. Accordingly, animal cells tightly monitor and maintain PM integrity.

Extracellular calcium (Ca^2+^) is essential for PM repair (5). Upon PM damage, Ca^2+^ flows from the extracellular space into the cytoplasm and initiates the recruitment of membrane repair factors to the lesion site (1). Annexins are cytosolic proteins that bind phospholipids in the presence of Ca^2+^ and participate in various aspects of PM repair, including membrane patching and tension reduction (1, 6, 7). Consistent with an important role in PM repair, genetic depletion of annexins in cultured cells impairs membrane resealing (8–12), and loss of annexin expression or function in mice causes muscular dystrophy (13–15).

In addition to annexins, the endosomal sorting complex required for transport (ESCRT) machinery promotes PM repair (16). The core ESCRT machinery, consisting of ESCRT-0, ESCRT-I, ESCRT-II, ESCRT-III, ALIX, and Vps4, were originally identified for their role in sorting ubiquitylated membrane proteins into the intraluminal vesicles of multivesicular bodies (17, 18). However, it is now clear that the ESCRT machinery mediates reverse-topology membrane scission in diverse cellular processes including nuclear envelope sealing, autophagosome closure, cytokinesis, viral budding, and lysosome repair (19–21). Interestingly, ESCRT I-III are recruited to damaged lysosomes (22, 23), whereas only ESCRT-III is recruited to PM lesions (24). Upon PM disruption, annexin A7 (ANXA7) localizes to the membrane lesion and recruits ALG-2, which subsequently recruits ESCRT-III (25). However, recent studies have suggested that ALG-2 can directly bind membranes in the presence of Ca^2+^ (26, 27). Thus, the extent to which annexin recruitment is coupled to ESCRT-III assembly during PM repair remains unclear.

Mutations in annexin A11 (ANXA11) and CHMP2B (a member of ESCRT-III) have been linked to amyotrophic lateral sclerosis (ALS), frontotemporal dementia (FTD), and limb-girdle muscular dystrophy (28–31), and a recent report has indicated that these mutations compromise PM repair (32). Given increasing evidence that PM repair is compromised in neurodegeneration and certain types of muscular dystrophy, we sought to employ complementary biochemical and genetic approaches to identify novel factors required for this essential membrane repair pathway.

## Results

### Identification of Proteins Recruited to the Inner Leaflet of the PM Upon Ca^2+^ Influx.

**Fig. 1.**
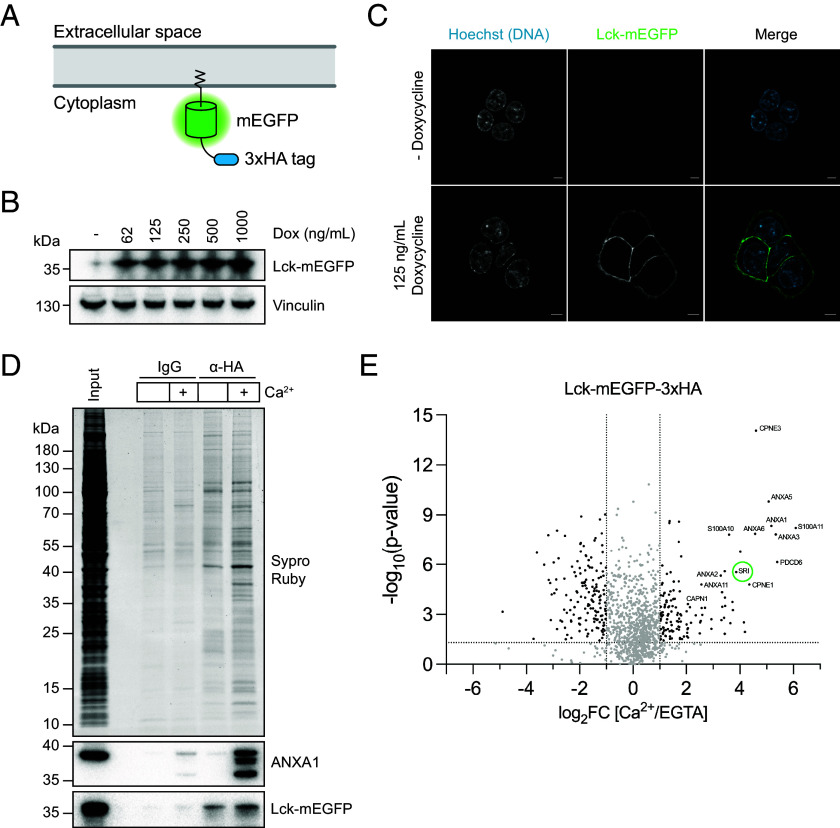
Quantitative organellar proteomics identifies Ca^2+^-dependent PM-binding proteins. (*A*) Schematic illustrating the membrane topology of Lck-mEGFP-3xHA when inserted into the PM. (*B*) Immunoblot analysis of HCT116 Lck-mEGFP-3xHA cells treated with increasing concentrations of doxycycline for 24 h. (*C*) Airyscan microscopy of HCT116 Lck-mEGFP-3xHA cells treated with vehicle or 125 ng/mL doxycycline for 24 h. Cyan: DNA (Hoechst 33342); Green: Lck-mEGFP-3xHA. (Scale bar, 5 µm.) (*D*) Total protein (Sypro Ruby staining) analysis of postnuclear supernatant input and IgG and anti-HA immunoprecipitants in the absence or presence of 1 mM free Ca^2+^. (*E*) Volcano plot showing proteins enriched in the anti-HA immunoprecipitants in the presence of Ca^2+^. Sorcin (SRI) is circled in green. Statistical significance was calculated using an unpaired two-tailed Student’s *t* test. See corresponding Dataset S1.

Ca^2+^ influx upon PM disruption recruits a repertoire of membrane repair proteins to the lesion site and is required for repair (2). We sought to leverage these features of PM repair to identify new repair factors. We designed a membrane fractionation strategy that would allow us to identify proteins that are recruited to the surface of inside-out PM vesicles in the presence of Ca^2+^, reasoning that some of these factors might promote PM repair. We generated an HCT116 cell line that inducibly expressed monomeric enhanced green fluorescent protein (mEGFP) fused to the PM targeting sequence of Lck and a 3xHA epitope tag (Lck-mEGFP-3xHA) (Fig. 1*A*). Incubation of these cells with doxycycline induced titratable expression of Lck-mEGFP-3xHA (Fig. 1*B*), and visualization of these cells after doxycycline induction revealed specific labeling of the PM with mEGFP (Fig. 1*C*).

To identify proteins that were recruited to the PM inner leaflet upon Ca^2+^ influx, we induced Lck-mEGFP-3xHA expression, mechanically ruptured cells, and performed IgG or anti-HA immunoprecipitations in the absence or presence of Ca^2+^. We observed the recruitment of multiple proteins to the surface of inside-out PM vesicles upon addition of Ca^2+^ (Fig. 1*D*). Annexin A1 (ANXA1) served as a positive control and was greatly enriched in the anti-HA + Ca^2+^ immunoprecipitant. Label-free quantitative mass spectrometry analysis of the anti-HA immunoprecipitants identified many proteins that were reproducibly recruited to the inner leaflet of the PM in the presence of Ca^2+^ (Fig. 1*E* and Dataset S1). Many significant hits identified in our mass spectrometry analysis (such as annexins, S100A10/11, ALG-2, and calpains) have been previously implicated in PM repair. These data confirmed that our inside-out PM vesicle isolation strategy faithfully recapitulated the Ca^2+^-dependent recruitment of repair factors to the PM inner leaflet, validating its utility for identifying new PM repair factors.

### Genome-Wide CRISPR Interference Screens for PM Repair.

**Fig. 2.**
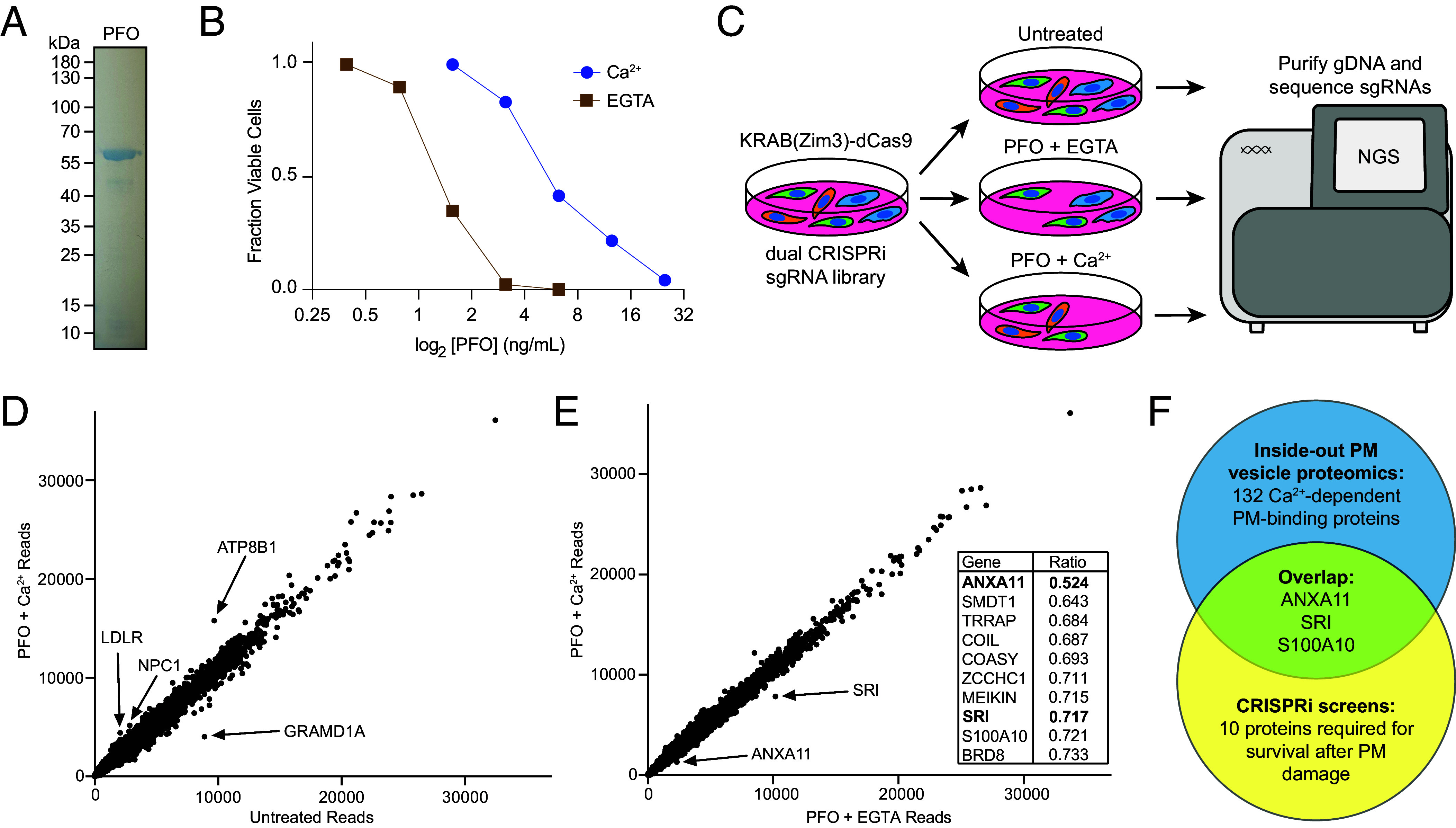
Genome-wide CRISPR interference (CRISPRi) screens identify genes required for survival after PM damage. (*A*) Coomassie-stained SDS-PAGE gel showing the purification of recombinant perfringolysin O (PFO). (*B*) Quantification of cell viability, as measured by trypan blue exclusion, plotted as a function of PFO concentration. (*C*) Schematic of the three genome-wide CRISPRi screen conditions. PFO doses that killed 25% of cells were used for the genetic screens. (*D*) Gene enrichment comparing untreated (2 mM extracellular Ca^2+^) and PFO + 2 mM extracellular Ca^2+^ screens revealed factors involved in PFO binding and cholesterol trafficking. See corresponding Dataset S2. (*E*) Gene enrichment comparing PFO + 2 mM extracellular EGTA and PFO + 2 mM extracellular Ca^2+^ screens identified Ca^2+^-dependent PM repair factors. *Inset*: top 10 genes depleted under PFO + 2 mM extracellular Ca^2+^ treatment. See corresponding Dataset S2. (*F*) Venn diagram showing common hits between the inside-out PM vesicle proteomics (Fig. 1*E*) and the CRISPRi screens (Fig. 2*E*).

Simultaneously, we performed complementary genetic screens to identify genes whose depletion sensitized cells to PM damage inflicted by treatment with the pore-forming toxin perfringolysin O (PFO). To achieve this, we first purified recombinant PFO from bacterial cells (Fig. 2*A*). Consistent with a requirement for extracellular Ca^2+^ in PM repair, an approximately fivefold lower PFO dose was sufficient to kill cells in the absence of extracellular Ca^2+^ (Fig. 2*B*). Exploiting this sensitization, we designed a genome-wide CRISPR interference (CRISPRi) survival screen strategy to distinguish likely PM repair factors from genes that affect PFO binding (Fig. 2*C*). Library-transduced cells were treated with PFO at doses that killed 25% of cells in the presence or absence of extracellular Ca^2+^ (Fig. 2*B*). CRISPRi guide RNA (sgRNA) enrichment was determined by deep sequencing of PCR-amplified barcodes (Dataset S2) (33).

When comparing the untreated and PFO + Ca^2+^ screens, the most enriched CRISPRi sgRNAs corresponded to genes involved in PFO binding and cholesterol trafficking (Fig. 2*D*). PFO is a cholesterol-dependent cytolysin that recognizes cholesterol in the outer leaflet of the PM (34). In contrast, comparison between the PFO + EGTA (Ca^2+^ chelator) and the PFO + Ca^2+^ screens revealed several Ca^2+^-binding proteins (Fig. 2 *E*, *Inset*).

Comparison of the hits identified in our proteomic and genetic screens revealed 3 overlapping genes: annexin A11 (ANXA11), sorcin (SRI), and S100A10 (Fig. 2*F*). ANXA11 and S100A10 have been previously implicated in PM repair. ALS-linked mutations in ANXA11 affect ESCRT-III dynamics at the PM (32). We previously showed that a S100A10-annexin A2 (ANXA2) complex localizes to PM lesions before being cleaved by calpains (35). Of particular interest, sorcin, uncharacterized in the context of membrane repair, emerged as a hit in both our proteomic and genetic screens (Fig. 2*F*).

### Sorcin Is Recruited to PM Lesions Upon Ca^2+^ Influx and Required for Membrane Repair.

**Fig. 3.**
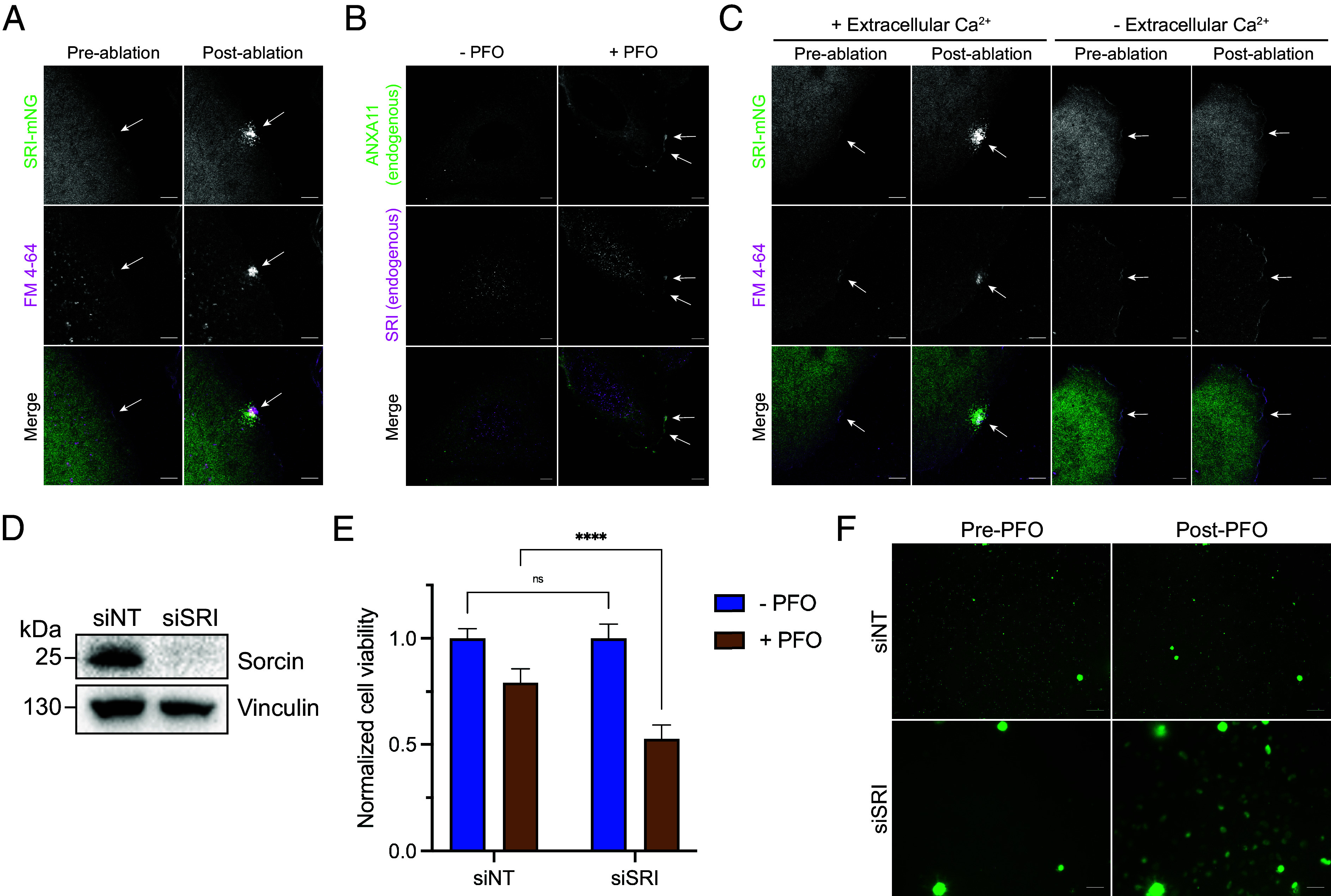
Sorcin is recruited to PM lesions upon Ca^2+^ influx and required for membrane repair. (*A*) Representative confocal micrographs of U2-OS Sorcin-mNeonGreen (SRI-mNG) cells subjected to laser ablation wounding. Images shown are pre- and postlaser ablation. Green: SRI-mNG; Magenta: FM4-64. (Scale bar, 5 µm.) See corresponding Movie S1. (*B*) Immunofluorescence microscopy of endogenous ANXA11 and SRI from U2-OS cells treated with vehicle or perfringolysin O (PFO; 25 ng/mL) for 5 min. Green: ANXA11; Magenta: SRI. (Scale bar, 5 µm.) (*C*) Representative confocal micrographs of U2-OS SRI-mNG cells subjected to laser ablation wounding in the presence or absence of 2 mM extracellular Ca^2+^. Images shown are pre- and postlaser ablation. Green: SRI-mNG; Magenta: FM4-64. (Scale bar, 5 µm.) See corresponding Movies S2 and S3. (*D*) Immunoblot analysis of U2-OS wild-type cells transfected with control (siNT) or sorcin-targeting (siSRI) siRNAs. (*E*) Normalized cell viability of U2-OS siNT and siSRI cells following treatment with vehicle or PFO (25 ng/mL). The graph displays mean ± SD (n = 9). Statistical significance was calculated using a two-way ANOVA (ns = not significant, ****P < 0.0001). (*F*) Representative confocal micrographs of U2-OS siNT and siSRI cells treated with PFO (25 ng/mL) in the presence of SYTOX Green (2.5 µM). Images shown are pre- and post-PFO treatment. Green: SYTOX Green. (Scale bar, 50 µm.)

Sorcin, like ALG-2, is a member of the Ca^2+^-binding penta-EF-hand (PEF) family of proteins (36). Sorcin was initially identified in multidrug-resistant cancer cells and has been suggested to regulate intracellular Ca^2+^ homeostasis (37–42). Despite this, a role for sorcin in membrane repair has not been described. To determine whether sorcin was a bona fide PM repair factor, we first tested whether sorcin is recruited to PM lesions. We generated a U2-OS cell line expressing fluorescently tagged sorcin (SRI-mNG) to monitor sorcin dynamics after laser ablation wounding. FM4-64 is a membrane-impermeable dye that brightly labels the site of damage and the PM repair cap. Upon laser ablation, we observed FM4-64 staining and sorcin recruitment at the site of PM damage (Fig. 3*A* and Movie S1). We next tested whether endogenous sorcin is recruited to PM lesions. Immunofluorescence microscopy of vehicle- and PFO-treated cells revealed that endogenous sorcin is recruited to sites of PM damage (Fig. 3*B*). Because extracellular Ca^2+^ is essential for PM repair (5), we asked whether Ca^2+^ influx was required for sorcin recruitment to PM lesions. Laser ablation experiments revealed that the removal of extracellular Ca^2+^ completely blocked both PM repair cap formation and sorcin recruitment to PM lesions (Fig. 3*C* and Movies S2 and S3).

We then asked whether sorcin depletion sensitized cells to PM damage. To address this, we depleted sorcin by RNA interference (Fig. 3*D*) and treated control (siNT) and sorcin-depleted (siSRI) cells with PFO (Fig. 3*E*). We observed that sorcin depletion sensitized cells to PFO treatment. We then tested whether sorcin depletion inhibited PM repair by monitoring the influx of the membrane-impermeable dye SYTOX Green into siNT and siSRI cells upon PFO treatment. Measurement of SYTOX Green influx revealed that sorcin depletion compromised PM repair (Fig. 3*F*). Together, these experiments established sorcin as a bona fide PM repair factor.

### Sorcin Simultaneously Binds ANXA11 and ALIX in the Presence of Ca^2+^.

Prompted by our observation that Ca^2+^ influx is essential for sorcin recruitment to PM lesions (Fig. 3*C*), we sought to identify proteins that interact with sorcin in the presence of Ca^2+^. Sorcin was previously reported to interact with a recombinant N-terminal fragment of ANXA11 (43), the strongest Ca^2+^-dependent candidate identified in our CRISPRi screens for cell survival upon PFO challenge (Fig. 2*E*). Additionally, ALG-2 has been demonstrated to bind ALIX (44). Motivated by these observations, we hypothesized that sorcin associates with ANXA11 and components of the ESCRT-III complex in the presence of Ca^2+^.

**Fig. 4.**
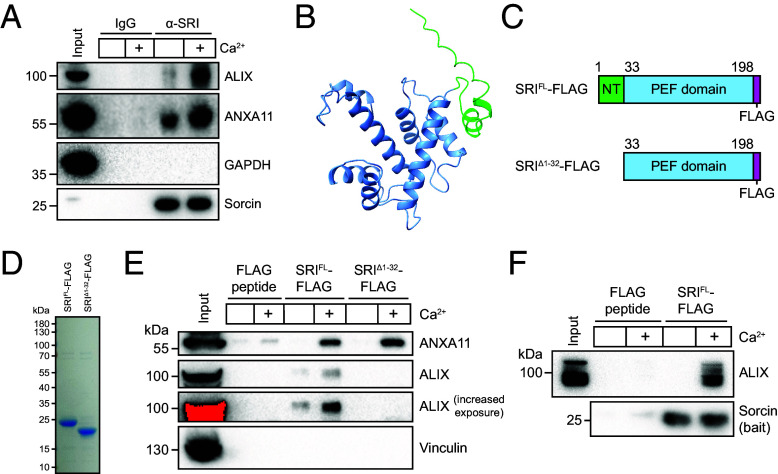
Sorcin simultaneously binds ANXA11 and ALIX in the presence of Ca^2+^. (*A*) Immunoblot analysis of postnuclear supernatant input and IgG and antisorcin immunoprecipitants in the absence or presence of 1 mM free Ca^2+^. (*B*) Alphafold3 predicted protein structure of human sorcin (SRI). The flexible N-terminal domain (NT) is colored green, and the penta-EF-hand (PEF) domain is colored blue. (*C*) Domain architecture of full-length (SRI^FL^-FLAG) and truncated (SRI^Δ1-32^-FLAG) sorcin constructs. (*D*) Coomassie-stained SDS-PAGE gel showing the purification of SRI^FL^-FLAG and SRI^Δ1-32^-FLAG. (*E*) Immunoblot analysis of cytosol input and FLAG peptide, SRI^FL^-FLAG, and SRI^Δ1-32^-FLAG immunoprecipitants in the absence or presence of 1 mM free Ca^2+^. (*F*) Immunoblot analysis of recombinant protein input and immunoprecipitants from an SRI^FL^-FLAG/ALIX in vitro binding assay in the absence or presence of 1 mM free Ca^2+^.

To test this hypothesis, we first performed immunoprecipitation experiments to evaluate whether endogenous sorcin associates with ANXA11 and ALIX. Immunoblot analysis of IgG and antisorcin immunoprecipitants revealed that endogenous sorcin associates with ANXA11 and ALIX in the presence of Ca^2+^ (Fig. 4*A*). We then sought to determine which domain of sorcin mediates its interaction with ANXA11 and ALIX. Alphafold3 modeling revealed that sorcin consists of two domains: a flexible N-terminal domain (amino acids 1 to 32) and a PEF domain (amino acids 33 to 198) (Fig. 4*B*). Given previous evidence indicating that the N terminus of PEF proteins regulates their association with other proteins (45), we purified FLAG-tagged full-length (SRI^FL^-FLAG) and truncated (SRI^Δ1-32^-FLAG) sorcin from bacterial cells (Fig. 4 *C* and *D*) for immunoprecipitation experiments. Full-length and truncated sorcin were immobilized on anti-FLAG beads, incubated with cytosol in the absence or presence of Ca^2+^, and analyzed by immunoblotting (Fig. 4*E*). These experiments revealed that sorcin bound to ANXA11 and ALIX in the presence of Ca^2+^. Notably, truncated sorcin retained its ability to bind ANXA11, but not ALIX. We then asked whether sorcin directly associates with ALIX. *In vitro* binding assays with purified recombinant proteins revealed that sorcin directly binds ALIX in the presence of Ca^2+^ (Fig. 4*F*). Together, these data demonstrated that sorcin simultaneously binds ANXA11 and ALIX in the presence of Ca^2+^.

### ANXA11 Is Required for Membrane Repair and Sorcin Recruitment to PM Lesions.

**Fig. 5.**
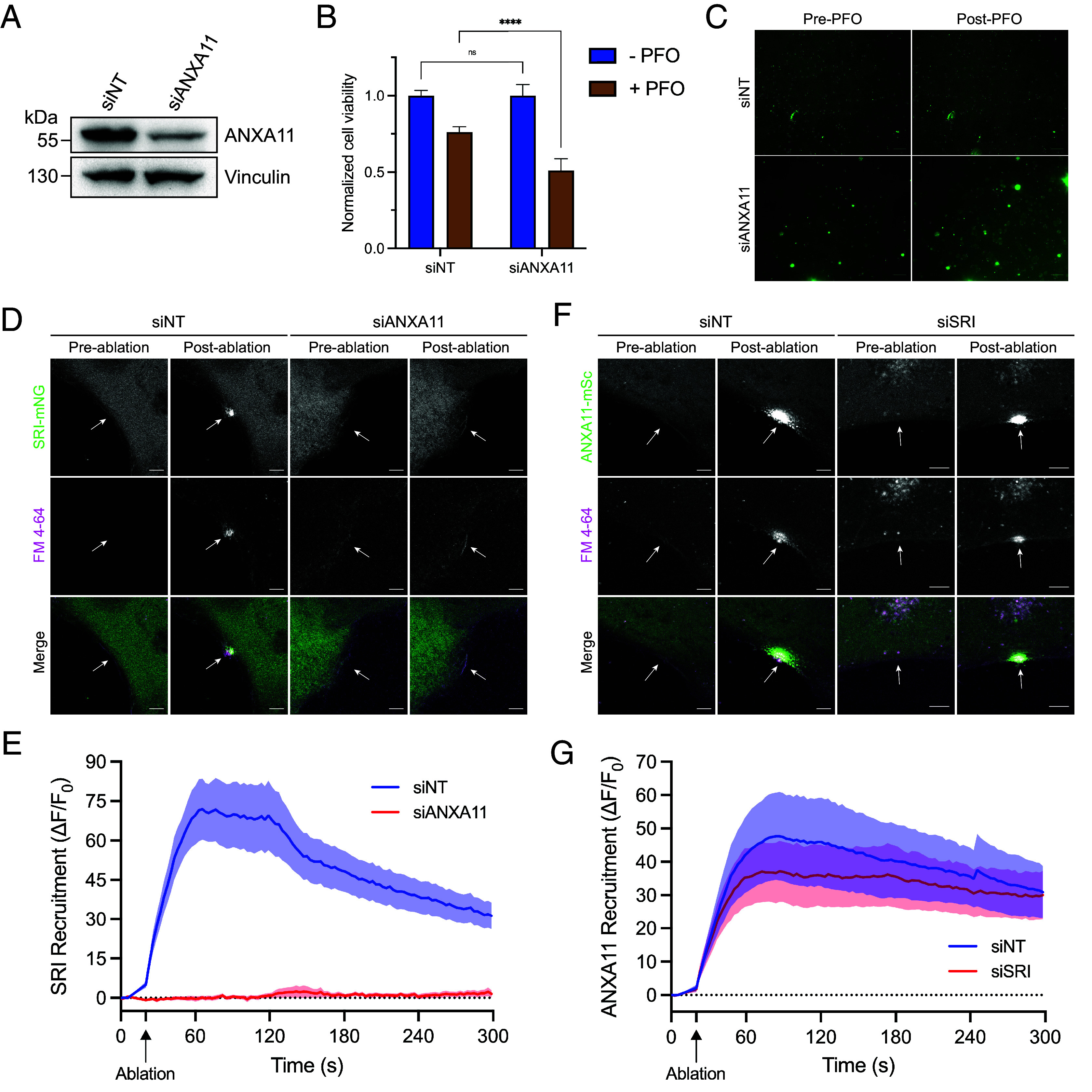
ANXA11 is required for PM repair and sorcin recruitment to PM lesions. (*A*) Immunoblot analysis of U2-OS wild-type cells transfected with control (siNT) or ANXA11-targeting (siANXA11) siRNAs. (*B*) Normalized cell viability of U2-OS siNT and siANXA11 cells following treatment with vehicle or perfringolysin O (PFO; 25 ng/mL). The graph displays mean ± SD (n = 6). Statistical significance was calculated using a two-way ANOVA (ns = not significant, ****P < 0.0001). (*C*) Representative confocal micrographs of U2-OS siNT and siANXA11 cells treated with PFO (25 ng/mL) in the presence of SYTOX Green (2.5 µM). Images shown are pre- and post-PFO treatment. Green: SYTOX Green. (Scale bar, 50 µm.) (*D*) Representative confocal micrographs of U2-OS Sorcin-mNeonGreen (SRI-mNG) siNT and siANXA11 cells subjected to laser ablation wounding. Images shown are pre- and postlaser ablation. Green: SRI-mNG; Magenta: FM4-64. (Scale bar, 5 µm.) See corresponding Movies S4 and S5. (*E*) Quantification of the recruitment dynamics of SRI-mNG cells transfected with siNT (n = 19) or siANXA11 (n = 14) siRNAs. The graph displays mean ± SEM. (*F*) Representative confocal micrographs of U2-OS ANXA11-mScarlet (ANXA11-mSc) siNT and sorcin knockdown (siSRI) cells subjected to laser ablation wounding. Images shown are pre- and postlaser ablation. Green: ANXA11-mSc; Magenta: FM4-64. (Scale bar, 5 µm) See corresponding Movies S6 and S7. (*G*) Quantification of the recruitment dynamics of ANXA11-mSc cells transfected with siNT (n = 9) or siSRI (n = 13) siRNAs. The graph displays mean ± SEM.

Our sorcin truncation data led us to hypothesize that sorcin couples ANXA11 recruitment and ESCRT-III assembly during PM repair. We thus evaluated the role of ANXA11 in PM repair and sorcin recruitment to PM lesions. We first tested whether ANXA11 was required for PM repair. We depleted ANXA11 by RNA interference (Fig. 5*A*) and treated siNT and ANXA11-depleted (siANXA11) cells with PFO (Fig. 5*B*). This experiment revealed that ANXA11 depletion sensitized cells to PFO. We then performed SYTOX Green influx experiments in siNT and siANXA11 cells and found that ANXA11 depletion compromised PM repair (Fig. 5*C*).

We then asked whether ANXA11 is required for sorcin recruitment to PM lesions. Laser ablation studies revealed that ANXA11 depletion blocked sorcin recruitment to PM lesions (Fig. 5 *D* and *E* and Movies S4 and S5). In contrast, sorcin depletion did not affect ANXA11 recruitment to PM lesions (Fig. 5 *F* and *G* and Movies S6 and S7). Notably, PM repair cap formation (as indicated by FM4-64 staining) was defective in ANXA11-depleted cells (Fig. 5*D*).

### Sorcin and ANXA11 Are Required for ESCRT-III Recruitment to PM Lesions.

**Fig. 6.**
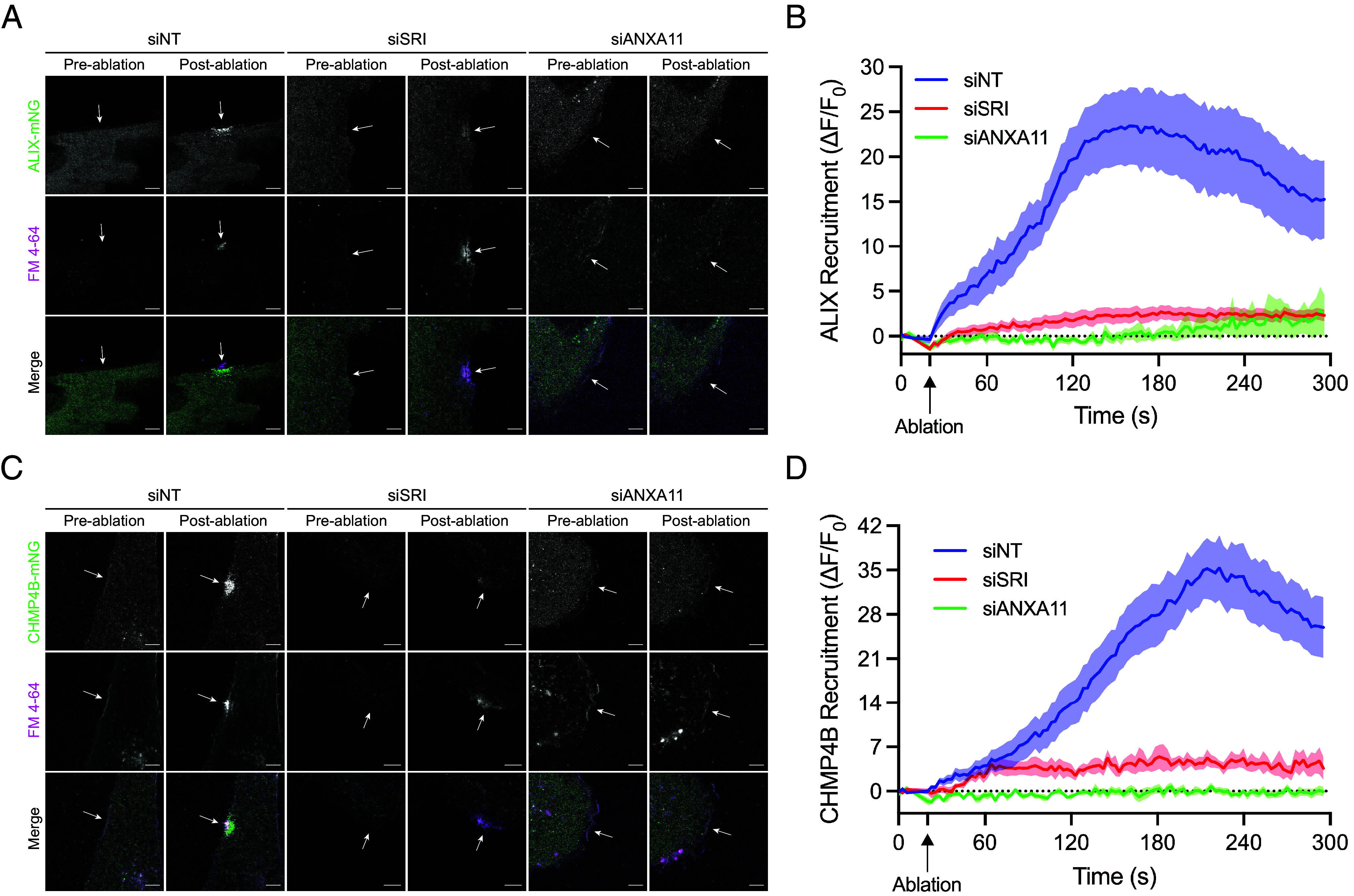
Sorcin and ANXA11 are required for ESCRT-III recruitment to PM lesions. (*A*) Representative confocal micrographs of U2-OS ALIX-mNeonGreen (ALIX-mNG) control (siNT), sorcin knockdown (siSRI), and ANXA11 knockdown (siANXA11) cells subjected to laser ablation wounding. Images shown are pre- and postlaser ablation. Green: ALIX-mNG; Magenta: FM4-64. (Scale bar, 5 µm.) See corresponding Movies S8–S10. (*B*) Quantification of the recruitment dynamics of ALIX-mNG cells transfected with siNT (n = 15), siSRI (n = 12), or siANXA11 (n = 11) siRNAs. The graph displays mean ± SEM. (*C*) Representative confocal micrographs of U2-OS CHMP4B-mNeonGreen (CHMP4B-mNG) siNT, siSRI, and siANXA11 cells subjected to laser ablation wounding. Images shown are pre- and postlaser ablation. Green: CHMP4B-mNG; Magenta: FM4-64. (Scale bar, 5 µm.) See corresponding Movies S11–S13. (*D*) Quantification of the recruitment dynamics of CHMP4B-mNG cells transfected with siNT (n = 15), siSRI (n = 7) or siANXA11 (n = 7) siRNAs. The graph displays mean ± SEM.

After establishing roles for ANXA11 in PM repair and sorcin recruitment to PM lesions, we asked whether sorcin and ANXA11 were required for ESCRT-III recruitment. To address this, we depleted sorcin or ANXA11 in U2-OS cells expressing fluorescent ALIX and CHMP4B fusion proteins and performed laser ablation studies. Strikingly, depletion of either sorcin or ANXA11 blocked the recruitment of both ALIX (Fig. 6 *A* and *B* and Movies S8–S10) and CHMP4B (Fig. 6 *C* and *D* and Movies S11–S13) to PM lesions.

### Sorcin Is Recruited to PM Lesions Together With ANXA11 and Before ESCRT-III.

**Fig. 7.**
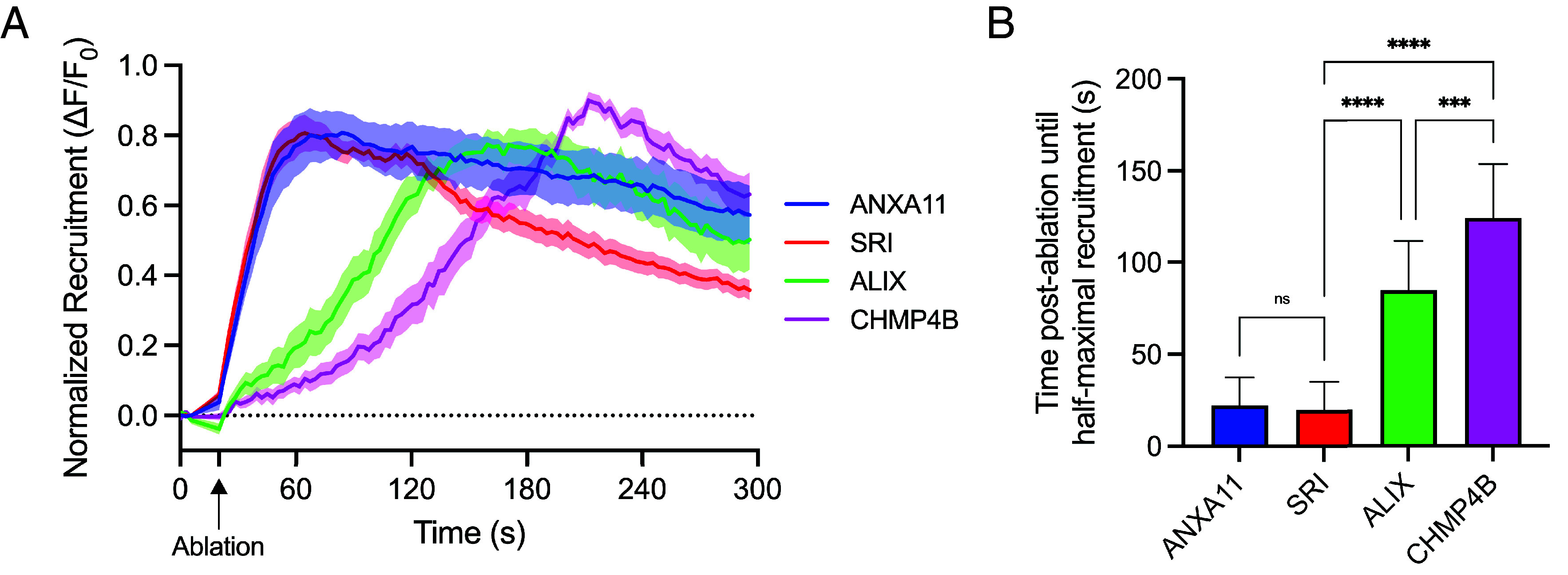
Sorcin is recruited to PM lesions together with ANXA11 and before ESCRT-III. (*A*) Quantification of the recruitment dynamics of ANXA11-mSc (n = 9), Sorcin-mNG (n = 19), ALIX-mNG (n = 15), and CHMP4B-mNG (n = 15) cells transfected with a nontargeting siRNA (siNT) from Figs. 5 *E* and *G* and 6 *B* and *D*. The graph displays mean ± SEM. (*B*) Quantification of the time postablation until half-maximal recruitment of ANXA11-mSc (n = 9), Sorcin-mNG (n = 19), ALIX-mNG (n = 15), and CHMP4B-mNG (n = 15) siNT cells from Figs. 5 *E* and *G* and 6 *B* and *D*. The graph displays mean ± SD. Statistical significance was calculated using a one-way ANOVA (ns = not significant, ***P < 0.001, ****P < 0.0001).

We then sought to establish the chronology in which ANXA11, sorcin, ALIX, and CHMP4B are recruited to sites of PM damage. We reanalyzed our laser ablation data (Figs. 5 and 6) and quantified the recruitment kinetics of each protein at PM lesions (Fig. 7 *A* and *B*). This analysis revealed that ANXA11 and sorcin are simultaneously recruited to PM lesions, followed sequentially by ALIX and then CHMP4B. These data, taken together with our truncation (Fig. 4) and laser ablation (Figs. 5 and 6) data, suggested a model in which ANXA11 anchors at PM lesions to facilitate sorcin recruitment and subsequent ESCRT-III assembly for PM repair.

### ANXA11 Is Sufficient to Recruit Sorcin to Liposomes in the Presence of Ca^2+^.

**Fig. 8.**
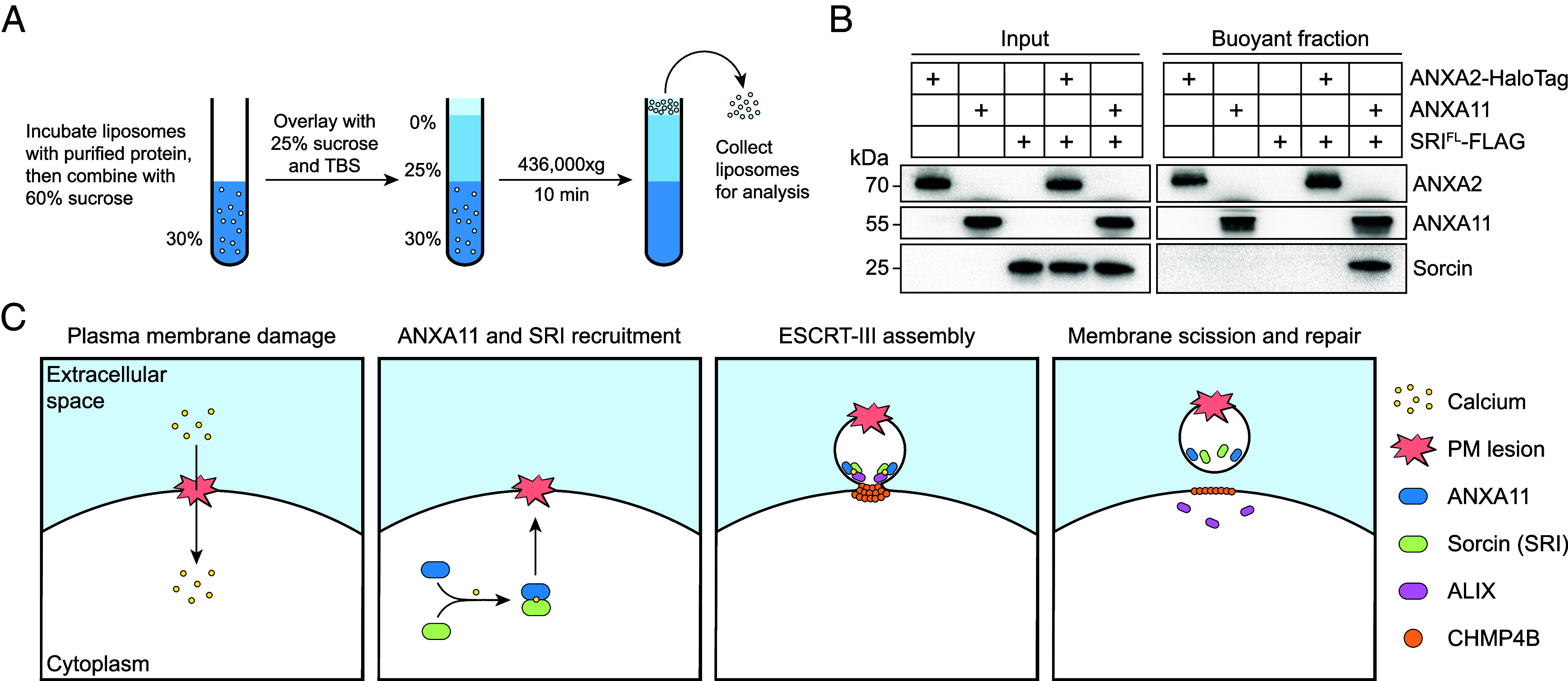
ANXA11 is sufficient to recruit sorcin to liposomes in the presence of Ca^2+^. (*A*) Schematic of the liposome flotation assay. Purified proteins were incubated with synthetic liposomes and 2 mM free Ca^2+^ prior to flotation through a sucrose density gradient to determine which proteins associated with membranes. (*B*) Immunoblot analysis of the liposome flotation assays. The components of each reaction are indicated. (*C*) Schematic depicting the current working model of sorcin-mediated PM repair.

We directly tested a part of this model by performing liposome flotation assays with purified components (Fig. 8*A*). Recombinant sorcin was incubated with synthetic liposomes and Ca^2+^ in the absence or presence of recombinant ANXA2 or ANXA11. The binding reactions were applied to the bottom of sucrose density gradients followed by high-speed centrifugation. Buoyant liposomes and bound proteins were solubilized then evaluated by immunoblot analysis. Sorcin alone was unable to float with liposomes, indicating that sorcin cannot directly bind membranes (Fig. 8*B*). However, sorcin floated with liposomes when ANXA11, but not ANXA2, was added to the binding reaction. These results indicated that sorcin binds directly and specifically to ANXA11 and that ANXA11 is sufficient to recruit sorcin to membranes in the presence of Ca^2+^ (Fig. 8*B*).

## Discussion

The molecular basis by which PM damage sensing is coupled to ESCRT-III assembly remains incompletely understood. Here, we identify sorcin as an early-acting Ca^2+^-dependent scaffold that bridges ANXA11-mediated PM damage sensing and ESCRT-III assembly at sites of membrane damage (Fig. 8*C*). Our data support a model in which ANXA11, recruited to the PM upon damage-induced Ca^2+^ influx, serves as an anchor that promotes the sequential recruitment of sorcin and ESCRT-III at PM lesions (Figs. 5–8). The direct and simultaneous engagement of sorcin with ANXA11 and ALIX via its PEF domain and flexible N terminus, respectively, provides a molecular explanation for how annexin recruitment is coupled to ESCRT-III assembly during PM repair (Fig. 4*E*). Genetic depletion of sorcin sensitizes cells to PM damage and compromises ESCRT-III recruitment, but not ANXA11 recruitment, to PM lesions (Figs. 3 *D*–*F*, 5 *F* and *G*, and 6), establishing sorcin as a critical PM repair factor.

ANXA11 depletion substantially decreases the size of the FM4-64-positive repair cap (Fig. 5*D*), consistent with our previous finding that ANXA2 depletion has a similar effect (35). Together, these data suggest that annexins play nonredundant roles in repair cap formation and that the proper balance of annexins is required for successful repair cap assembly. Furthermore, sorcin binds to the proline-rich ANXA11 N terminus, which resembles the motif that we previously found to be cleaved by calpains during PM repair. Thus, calpains may regulate ANXA11-mediated ESCRT-III recruitment.

The ANXA11/SRI pathway may synergize with the reported ANXA7/ALG-2 pathway to recruit ESCRT-III to PM lesions (25). However, we have found some biochemical differences between sorcin and ALG-2. Our data indicate that sorcin simultaneously engages ANXA11 and ALIX via its flexible N terminus and PEF domain, respectively. Additionally, sorcin is unable to directly bind membranes in the presence of Ca^2+^. In contrast, ALG-2 interacts with both ANXA7 and ALIX via its PEF domain (46, 47) and has recently been reported to directly bind membranes (26, 27). Thus, our data establish a mechanism by which annexin recruitment is directly coupled to ESCRT-III assembly during PM repair.

Genetic mutations in ESCRT machinery components have been implicated in various neurodegenerative diseases and membrane repair disorders (28, 48–52). In addition, ANXA11 mutations have been associated with ALS and muscular dystrophy (29–31). Although these mutations have been studied primarily in the context of lysosomal membrane repair, a recent report found that ALS- and FTD-associated mutations in ANXA11 and CHMP2B also compromise PM repair (32). Our identification of sorcin as a Ca^2+^-dependent scaffold that recruits ESCRT-III to PM lesions via ANXA11 raises the possibility that disease-associated ANXA11 mutations might impair PM repair, at least in part, by disrupting sorcin recruitment to PM lesions. Consistent with this suggestion, multiple disease-associated ANXA11 mutations map to the same flexible N-terminal region that binds sorcin.

Finally, our data highlight mechanistic and topological similarities between the budding of membrane-enveloped viruses (e.g., HIV-1) and damage-induced PM vesicles (microvesicles). Göttlinger and colleagues provided the initial piece of evidence that host factors contribute to virus budding (53). Subsequent work by the Sundquist and Göttlinger teams revealed that the p6 region of the HIV-1 Gag protein engages TSG101 and ALIX, which subsequently recruit ESCRT-III and Vps4 for membrane constriction and scission (54–56). More recently, we found that PM damage elicits extracellular vesicle secretion and that expression of a dominant-negative Vps4 mutant, which blocks ESCRT-III disassembly and vesicle scission, reduces the secretion of damage-induced microvesicles (35, 57, 58). Our identification of sorcin as a membrane repair factor that localizes to PM lesions and recruits ESCRT-III for microvesicle shedding leads us to propose that enveloped viruses and PM damage-induced microvesicles share a common mechanism of membrane budding. Specifically, we propose that ANXA11 and sorcin function analogously to retroviral Gag proteins in recruiting ALIX and ESCRT-III to the PM for membrane budding. We speculate that membrane-enveloped viruses may have co-opted this host pathway of PM ESCRT recruitment to facilitate virion assembly and propagation.

## Materials and Methods

### Cell Lines, Media, and General Chemicals.

HEK293T, HCT116, MDA-MB-231, and U2-OS cell lines were cultured in 5% CO_2_ at 37 °C and maintained in Dulbecco’s Modified Eagle Medium (DMEM) supplemented with 10% fetal bovine serum (FBS). Cells were routinely tested and found negative for *Mycoplasma* contamination using the MycoAlert *Mycoplasma* Detection Kit (Lonza Biosciences). All cell lines used in this study were authenticated by the UC Berkeley Cell Culture Facility using STR profiling. Unless otherwise noted, all chemicals were purchased from Sigma-Aldrich.

### Antibodies.

Primary antibodies used for immunoblotting (1:1,000 dilution) were mouse anti-ALIX (Santa Cruz; sc-53540), rabbit anti-ALIX (Proteintech; 12422-1-AP), rabbit anti-annexin A1 (Abcam; ab214486), rabbit anti-annexin A2 (Abcam; ab185957), rabbit anti-annexin A11 (Proteintech; 10479-2-AP), rabbit anti-GAPDH (Cell Signaling Technology; 14C10) rabbit anti-HA-Tag (Cell Signaling Technology; C29F4), rabbit anti-SRI (Abcam; ab71983), and rabbit anti-vinculin (Abcam; ab129002). Secondary antibodies used for immunoblotting (1:10,000 dilution) were HRP-linked sheep anti-mouse IgG (Cytiva; NA931) and HRP-linked donkey anti-rabbit IgG (Cytiva; NA934).

Primary antibodies used for immunofluorescence (1:100 dilution) were mouse anti-sorcin (Santa Cruz; sc-100859) and rabbit anti-annexin A11 (Proteintech; 10479-2-AP). Secondary antibodies used for immunofluorescence (1:500 dilution) were Alexa Fluor 488 donkey anti-rabbit IgG (A-21202; Invitrogen) and Alexa Fluor 555 goat anti-mouse IgG (A-21424; Invitrogen).

### Plasmids.

Lck-mEGFP-3xHA was cloned into a pLIX403-Puro backbone. mNeonGreen and mScarlet fusion proteins were cloned into pLJM1-Puro and pLJM1-Blast backbones, respectively. Constructs for recombinant protein purification were cloned into a pET28a backbone. Recombinant PFO was appended at the C-terminus with a His_6_ tag. Recombinant full-length and truncated sorcin were appended at the C-terminus with a FLAG tag and a His_6_ tag. Recombinant ANXA2 was appended at the C-terminus with HaloTag and a His_6_ tag. All plasmid constructs were verified by whole-plasmid sequencing (ElimBio).

### Lentivirus Production and Transduction.

HEK293T cells at ~40% confluence in a 6-well plate were transfected with 165 ng of pMD2.G, 1.35 µg of psPAX2 and 1.5 µg of a lentiviral plasmid using 7.5 µL of TransIT-LT1 Transfection Reagent (Mirus Bio) according to the manufacturer’s protocol. For lentiviral expression of the genome-wide CRISPRi library, HEK293T cells at similar confluence within two 150 mm dishes were each transfected with 1 µg of pMD2.G, 7 µg of psPAX2, and 8 µg of the Dual sgRNA CRISPRi library (Addgene #187246) using 48 µL of TransIT-LT1 Transfection Reagent. The lentivirus-containing medium was harvested 48 h posttransfection by filtration through a 0.45 μm polyethersulfone filter (VWR Sciences). The filtered lentivirus was aliquoted, snap-frozen in liquid nitrogen, and stored at −80 °C until use. Cells were transduced with filtered lentivirus in the presence of 8 μg/mL polybrene for 24 h after which the medium was replaced. Cells were selected using 1 μg/mL puromycin, 5 µg/mL blasticidin, or 100 µg/mL hygromycin B.

### Immunoblotting.

Cells were lysed in Tris-buffered saline (TBS) supplemented with 1% Triton X-100 (TX-100) and a protease inhibitor cocktail (1 mM 4-aminobenzamidine dihydrochloride, 1 µg/mL antipain dihydrochloride, 1 µg/mL aprotinin, 1 µg/mL leupeptin, 1 µg/mL chymostatin, 1 mM phenylmethylsulfonyl fluoride (PMSF), 50 µM N-tosyl-L-phenylalanine chloromethyl ketone, and 1 µg/mL pepstatin) and incubated on ice for 15 min. Whole cell lysates were centrifuged at 15,000×*g* for 10 min at 4 °C, and the postnuclear supernatant (PNS) was diluted with 6X Laemmli buffer (reducing) to a 1X final concentration. Samples were heated at 95 °C for 5 min and resolved in 4 to 20% acrylamide Tris-glycine gradient gels (Life Technologies). The resolved proteins were then transferred to polyvinylidene difluoride (PVDF) membranes (EMD Millipore).

The PVDF membranes were blocked for 30 min with 5% bovine serum albumin (BSA) in TBS supplemented with 0.1% Tween-20 (TBS-T) and incubated overnight with 1:1,000 dilutions of primary antibodies in 5% BSA in TBS-T. The membranes were washed three times with TBS-T, incubated for 1 h at room temperature with 1:10,000 dilutions of HRP-conjugated secondary antibodies (Cytiva Life Sciences) in 5% BSA in TBS-T, washed three times with TBS-T, and washed once with TBS. Proteins were detected using ECL2 or PicoPLUS reagent (Thermo Fisher Scientific).

### Inside-Out PM Vesicle Immunopurification and Mass Spectrometry.

HCT116 Lck-mEGFP-3xHA cells were grown to ~90% confluence in six 150 mm dishes. Lck-mEGFP-3xHA expression was induced by replacing the conditioned medium with DMEM supplemented with 10% FBS and 0.125 µg/mL doxycycline 24 h prior to the experiment. All subsequent manipulations were performed at 4 °C. Each 150 mm dish was washed once with 5 mL of cold PBS supplemented with 1 mM EGTA then harvested by scraping into 5 mL of cold PBS containing 1 mM EGTA. The cells were collected by centrifugation at 300×*g* for 5 min, and the supernatant was discarded. The cell pellets were resuspended in 2 vol of organelle lysis buffer (1X TBS, 1 mM EGTA, 5 mM TCEP, 25 µM Calpain-I inhibitor and a protease inhibitor cocktail (see immunoblotting section)). The cell suspension was mechanically lysed by passing through a 25-gauge needle until ~85% of cells were lysed as assessed by trypan blue exclusion. The lysed cells were centrifuged at 1,000×*g* for 15 min to sediment intact cells and nuclei, and the PNS was transferred to a 15 mL conical tube.

Beads from 200 µL of a magnetic Protein G Dynabead (Thermo Fisher Scientific) slurry were sedimented with a magnetic tube rack and resuspended in organelle lysis buffer. The bead slurry was divided evenly into four tubes and resedimented. The PNS was equally divided and divided between the four tubes. Tubes 1 and 2 received 5 µg of mouse IgG isotype antibody (Cell Signaling Technology G3A1), and tubes 3 and 4 received 5 µg of mouse anti-HA antibody (Proteintech 66006-2-Ig). Tubes 2 and 4 also received CaCl_2_ (2 mM final). The binding reactions were incubated on a rotating mixer for 15 min and washed three times with organelle lysis buffer. The wash buffer for tubes 2 and 4 was supplemented with 2 mM CaCl_2_. Each sample was eluted by heating at 55 °C for 5 min in 65 µL of elution buffer [50 mM TEAB and 0.1% Rapigest (Waters)]. An aliquot of each eluate (15 µL) was reserved for Sypro Ruby staining and immunoblot analysis. The remaining eluates from tubes 3 and 4 (three biological replicates each) were sent to the UC Berkeley Vincent J. Coates proteomics facility for label-free quantitative mass spectrometry. The mass spectrometry data were analyzed using PEAKS Studio.

### Genome-Wide CRISPRi Screens for PM Repair.

MDA-MB-231 cells expressing Zim3-KRAB-dCas9-P2A-Hygro (Addgene #188768) were transduced with the genome-wide CRISPRi library at a multiplicity of infection (MOI) of 0.2. After puromycin selection, nine 150 mm dishes of CRISPRi library cells at ~80% confluence were washed once with 10 mL PBS and dissociated using 5 mL of accutase per dish. The cell suspension was pooled in a 50 mL conical tube and centrifuged at 300×*g* for 4 min. The cells were resuspended in 12 mL of TBS, divided equally into 3 tubes, and adjusted to 2 million cells/mL in each tube. Each tube received CaCl_2_, EGTA and/or PFO to achieve the following final concentrations: 2 mM CaCl_2_ (untreated), 0.78 ng/mL PFO and 2 mM EGTA (PFO + EGTA), or 3.125 ng/mL PFO and 2 mM CaCl_2_ (PFO + Ca^2+^). Cells were mixed by gentle inversion and incubated at 37 °C for 20 min. Cells were sedimented at 300×*g* for 4 min and resuspended in 12 mL of conditioned medium. The cell suspensions were distributed equally into six 150 mm dishes per condition. After 36 h, the cells (~25 million cells per condition) were harvested with accutase and stored at −80 °C.

### Genomic DNA Isolation.

Cells from each CRISPRi screen condition were lysed in 5 mL of genomic DNA lysis buffer (20 mM Tris-HCl pH 8.0, 10 mM EDTA, 100 mM NaCl, and 0.5% SDS) in a 15 mL conical tube. DNA was extracted by sequential phenol:chloroform:isoamyl alcohol (25:24:1, pH 8.0) and chloroform extractions, each performed by mixing until an emulsion formed, incubating for 5 min, and centrifuging at 5,000×*g* for 1 h. Between extractions, 2.5 mL of TE buffer was added to each organic phase for re-extraction, and the aqueous phases were pooled. DNA was precipitated from each combined aqueous phase (~10 mL) by addition of sodium acetate (1 mL of 3 M, pH 5.2) and ethanol (20 mL) and stored overnight at 4 °C.

Each sample was recovered by centrifugation at 5,000×*g* at 4 °C for 1 h. The pellets were resuspended in the remaining supernatant (~1 mL), transferred to a 1.5 mL tube, and centrifuged at 20,000×*g* at 4 °C for 5 min. The pellets were washed twice with 1 mL of 70% ethanol by centrifugation at 15,000×*g* at room temperature for 5 min and allowed to air dry for 10 min.

The dried pellets were resuspended in 0.75 mL of 1X RNase ONE reaction buffer (Promega), and RNase ONE (3.75 µL; Promega) and RNase A (Thermo Fisher Scientific) were added and incubated at 37 °C for 6 h. DNA was reprecipitated by the addition of sodium acetate (75 µL of 3 M, pH 5.2) and ethanol (1.5 mL), stored overnight at 4 °C, and each sample was recovered as described above before final resuspension in 0.75 mL of 2.5 mM Tris-HCl pH 8.0.

### CRISPRi Library Preparation and Sequencing.

A PCR reaction was assembled with the following components: 20 µL of oJR441 reverse primer (100 µM stock), 20 µL of indexed forward primer (100 µM stock), 1 mL of NEBNext Ultra II Q5 MasterMix, and 200 µg of genomic DNA. Each reaction was adjusted to 2 mL with nuclease-free water and distributed into 100 µL aliquots. PCR amplification was performed as follows: 1) 98 °C for 30 s; 2) 98 °C for 10 s; 3) 63 °C for 75 s; 4) steps 2 to 3 repeated 21 times; 5) 72 °C for 5 min. PCR amplicons were purified using a 0.5 to 0.65X double SPRI size selection (Beckman Coulter). The libraries were spiked with 5% PhiX and sequenced on a NextSeq P2 100PE flow cell (400 million paired-end reads, 25 cycles) using custom sequencing primers.

- oJR441 reverse primer sequence: CAAGCAGAAGACGGCATACGAGATGCGGCCGGCTGTTTCCAGCTTAGCTCTTAAA
- Indexed forward primer sequence: AATGATACGGCGACCACCGAGATCTACACnnnnnnnnCGCGTATCCCTTGGAGAACCACC
- Read 1 primer sequence: CGCGTATCCCTTGGAGAACCACCTTGTTGG
- Read 2 primer sequence: GCGGCCGGCTGTTTCCAGCTTAGCTCTTAAAC
- Index: CCAACAAGGTGGTTCTCCAAGGGATACGCG

### CRISPRi Screen Analysis.

A custom MATLAB (MathWorks) script was used to count the number of reads aligning to both sgRNAs for each gene. Genes with read counts in the bottom 20^th^ percentile were excluded from analysis to remove genes whose depletion affected cell growth and viability.

### Live-Cell Microscopy and Laser Ablation.

Images were acquired using an LSM900 confocal microscope (ZEISS) using confocal mode, a 63× Plan-Apochromat, NA 1.40 objective, and a heated, CO_2_-controlled chamber. Cells were seeded in 35 mm collagen-coated glass-bottom dishes (MatTek) and imaged in Fluorobrite DMEM supplemented with 10% FBS and 2.5 µM FM4-64 (Biotium). For laser ablation experiments, a 1 µm × 1 µm square region of interest was positioned over the edge of a cell not adjacent to another cell and ablated for 100 iterations using all lasers at 100% power. Protein recruitment was quantified in Zen 3.1 (Zeiss) by measuring fluorescence intensities within a box that encapsulated the repair cap. Fluorescence intensities of an adjacent, nonablated area of the membrane was measured and used for a frame-by-frame background subtraction. Recruitment was plotted as the background-corrected change in fluorescence intensity divided by the fluorescence intensity at the damage site prior to ablation.

For the laser ablation experiments in Fig. 3*C*, the cells were washed once with TBS and imaged in TBS supplemented with 2.5 µM FM4-64 and either 2 mM CaCl_2_ or 2 mM EGTA.

### Immunofluorescence Microscopy.

U2-OS cells grown to ~70% confluence on 12 mm glass coverslips (Corning) were treated with vehicle or 25 ng/mL PFO for 5 min at 37 °C. The cells were washed once with PBS, fixed in 4% EM-grade paraformaldehyde (Electron Microscopy Science), washed three times with PBS, and permeabilized and blocked in IF blocking buffer (2% FBS and 0.02% saponin in PBS) for 30 min at room temperature. Coverslips were transferred to a humidity chamber, incubated with primary antibodies (1:100 dilution in IF blocking buffer) for 1 h at room temperature, washed three times with PBS, incubated with fluorophore-conjugated secondary antibodies (1:500 dilution in IF blocking buffer) for 1 h at room temperature, and washed three times with PBS. The coverslips were then mounted overnight in ProLong Gold antifade mountant with DAPI (Thermo Fisher Scientific) and sealed with clear nail polish. Images were acquired using an LSM900 confocal microscope using Airyscan 2 mode and a 63× Plan-Apochromat, NA 1.40 objective.

### RNA Interference.

U2-OS cells were suspended using accutase and transfected using Lipofectamine RNAiMAX (Thermo Fisher Scientific) according to the manufacturer’s instructions. The cell suspension was diluted in conditioned medium to a final concentration of 1.5 × 10^5^ cells/mL, 20 nM total siRNA and 0.3% (v/v) transfection reagent, dispensed into individual wells of a poly-D-lysine-coated six-well plate and incubated overnight at 37 °C. Conditioned medium was replaced after 18 to 24 h. At 48 h posttransfection, cells were suspended using accutase, reseeded at an appropriate density for laser ablation, cell viability, or SYTOX Green influx experiments, and incubated at 37 °C for an additional 24 h. The following siRNAs were purchased from Qiagen: ANXA11 (Hs_ANXA11_6), SRI (Hs_SRI_10), and a nontargeting control (1027310).

### Cell Viability Assays.

U2-OS cells were transfected with siRNAs as described above, seeded in a poly-D-lysine-coated 24-well plate and grown to 70% confluence. At 72 h posttransfection, cells were treated with vehicle or 25 ng/mL PFO for 30 min in 200 μL of Fluorobrite DMEM. Fluorobrite DMEM was replaced with 200 µL of fresh DMEM supplemented with 10% FBS, and the cells were allowed to recover for 1 h at 37 °C. After the recovery period, 200 µL of Fluorobrite DMEM supplemented with 0.5 mg/mL MTT reagent was added to each well and incubated at 37 °C for 1 h. The MTT-containing medium was removed, replaced with 200 µL of DMSO and incubated for 5 min. An aliquot of each DMSO suspension (100 µL) was transferred to a 96-well plate, and the absorbance was measured at 570 nm. Cell viability was calculated by subtracting the absorbance of a DMSO blank from each sample measurement.

### Measurement of PM Permeabilization by PFO.

U2-OS cells were transfected with siRNAs as described above, seeded in 35 mm collagen-coated glass-bottom dishes and grown to 70% confluence. At 72 h posttransfection, cells were washed with PBS, placed in a 37 °C, 5% CO_2_ chamber on an LSM900 confocal microscope system and incubated with Fluorobrite DMEM supplemented with 10% FBS, 25 ng/mL PFO, and 2.5 μM SYTOX Green. SYTOX Green uptake was measured using the 20× Plan-Apochromat, NA 0.8 objective with images acquired every 10 s for 10 min.

### Recombinant Protein Purification.

Recombinant proteins were purified as described in (59) with the following modifications. Recombinant PFO was expressed in *E. coli* Rosetta2(DE3)pLysS cells. A preculture (5 mL) was grown overnight at 37 °C and diluted into a 500 mL culture. The culture was incubated at 37 °C until the OD_600_ reached ~0.6, and protein expression was induced by addition of 50 µM IPTG overnight at 30 °C. The cells were harvested by centrifugation at 5,000×*g* for 10 min in a swing-bucket H-6000A rotor, and the cell pellet was stored at −80 °C until use. All subsequent manipulations were performed at 4 °C. The cell pellet was resuspended in 5 mL of Ni-NTA lysis buffer 1 (50 mM HEPES pH 7.4, 150 mM NaCl, 10 mM imidazole pH 7.4 and a protease inhibitor cocktail (see immunoblotting section)) and sonicated 8 times (5 s on, 10 s off, 15% amplitude). The lysate was clarified by centrifugation at 5,000×*g* for 10 min in a fixed-angle FIBERlite F21-8x50y rotor, and the supernatant was added to 1 mL of pre-equilibrated HisPur™ Ni-NTA Resin and incubated on a rotating mixer for 1 h. The beads were washed three times with Ni-NTA wash buffer 1 (50 mM HEPES pH 7.4, 250 mM NaCl, 10 mM imidazole pH 7.4) and eluted with Ni-NTA elution buffer 1 (50 mM HEPES pH 7.4, 150 mM NaCl, 300 mM imidazole pH 7.4). The eluate was diluted with dilution buffer (50 mM HEPES pH 7.4, 150 mM NaCl, 10 mM DTT) to a final protein concentration of 1 mg/mL, snap-frozen in liquid nitrogen and stored at −80 °C until use.

Recombinant sorcin and ANXA2 were expressed in *E. coli* Rosetta2(DE3)pLysS cells. Precultures (30 mL) were grown overnight at 37 °C and diluted into 3,000 mL cultures. The cultures were incubated at 37 °C until the OD_600_ reached ~0.6, and protein expression was induced by addition of 0.2 mM IPTG for 4 h at 37 °C. The cells were harvested by centrifugation at 5,000×*g* for 10 min in a swing-bucket H-6000A rotor, and cell pellets were stored at −80 °C until use. All subsequent manipulations were performed at 4 °C. The cell pellets were resuspended in 30 mL of Ni-NTA lysis buffer 2 (20 mM Tris-HCl pH 8.0, 300 mM NaCl, 10 mM imidazole pH 8.0, and a protease inhibitor cocktail (see immunoblotting section)) and sonicated 5 times (5 s on, 15 s off, 20% amplitude). Each lysate was clarified by centrifugation at 20,000×*g* for 15 min in a fixed angle FIBERlite F21-8x50y rotor, and the supernatant fractions were applied to gravity flow columns containing 1 mL of pre-equilibrated HisPur™ Ni-NTA Resin (Thermo Fisher Scientific). Each column was washed with three column volumes of Ni-NTA wash buffer 2 (20 mM Tris-HCl pH 8.0, 300 mM NaCl, 25 mM imidazole pH 8.0), eluted with Ni-NTA elution buffer 2 (20 mM Tris-HCl pH 8.0, 300 mM NaCl, 300 mM imidazole pH 8.0). Eluted protein at 0.5 mg/mL was aliquoted, snap-frozen in liquid nitrogen and stored at −80 °C until use. Recombinant His_6_-ANXA11 was purchased from Bio-Techne.

### Endogenous Sorcin Immunoprecipitation.

HCT116 wild-type cells were grown to ~90% confluence in four 150 mm dishes. All subsequent manipulations were performed at 4 °C. Each dish was washed once with 5 mL of cold PBS supplemented with 1 mM EGTA and harvested by scraping into 5 mL of cold PBS containing 1 mM EGTA. Cells were pooled, collected by centrifugation at 300×*g* for 5 min, and the supernatant was discarded. The cell pellet was resuspended in 2.5 mL of IP buffer (1X TBS, 1 mM EGTA, 0.5% TX-100 and a protease inhibitor cocktail (see immunoblotting section)) and incubated for 10 min. The lysed cells were centrifuged at 15,000×*g* for 10 min to sediment insoluble material, and the supernatant was transferred to a new tube. An aliquot (50 μL) of the supernatant was retained for an input measurement, and the remaining lysate was distributed equally to four tubes.

Tubes 1 and 2 received 5 µg of mouse IgG isotype antibody (Cell Signaling Technology G3A1), and tubes 3 and 4 received 5 µg of mouse antisorcin antibody (Santa Cruz; sc-100859). Tubes 2 and 4 also received CaCl_2_ (2 mM final). Pre-equilibrated Protein A/G agarose beads (50 μL) (Santa Cruz Biotechnology) were added to each tube, and the binding reactions were incubated on a rotating mixer overnight. The beads were then washed three times with an IP buffer. The wash buffer for tubes 2 and 4 was supplemented with 2 mM CaCl_2_. Each sample was eluted in 1X Laemmli buffer (nonreducing) for immunoblot analysis.

### FLAG Immunoprecipitation.

HCT116 wild-type cells were grown to ~90% confluence in ten 150 mm dishes. All subsequent manipulations were performed at 4 °C. Each dish was washed once with 5 mL of cold PBS supplemented with 1 mM EGTA and harvested by scraping into 5 mL of cold PBS containing 1 mM EGTA. Cells were pooled, collected by centrifugation at 300×*g* for 5 min, and the supernatant was discarded. The cell pellet was resuspended in 2 vol of organelle lysis buffer, and the cell suspension was mechanically lysed by passing through a 25-gauge needle until ~85% of cells were lysed as assessed by trypan blue exclusion. The lysed cells were centrifuged at 1,000×*g* for 15 min to sediment intact cells and nuclei, and the PNS was centrifuged at 100,000×*g* for 30 min in an SW55 rotor. The supernatant (cytosol fraction) was collected carefully without disturbing the membrane pellet and diluted 1:2 in an organelle lysis buffer.

In parallel, 150 µL of anti-FLAG agarose beads (Chromotek ffa-20) were sedimented at 1,000×*g* for 2 min and resuspended in 600 µL of organelle lysis buffer. The resuspended beads were divided equally into six tubes. FLAG (3x) peptide, SRI^FL^-FLAG, and SRI^Δ1-32^-FLAG baits (4 µM each) were added to anti-FLAG beads in a 200 µL reaction. Bait proteins were bound to the anti-FLAG beads for 1 h at 4 °C. The beads were washed three times with an organelle lysis buffer and sedimented at 1,000×*g* for 2 min. The purified cytosol was equally divided and dispensed between the six tubes. CaCl_2_ (2 mM final) was added to the indicated tubes. The binding reactions were incubated on a rotating mixer for 2 h. The beads were washed three times with an organelle lysis buffer. The wash buffer for the Ca^2+^ immunoprecipitations was supplemented with 2 mM CaCl_2_. Each sample was eluted in 1X Laemmli buffer (nonreducing) for immunoblot analysis.

### Liposome Production.

Liposomes were generated by mixing lipids in a glass vial at the following molar ratios: 38:20:20:3:0.5:18.5 DOPC:POPE:DOPS:PI(4,5)P_2_:TexasRed-PE:cholesterol (1 µmol total lipid) in 500 µL of 5% methanol and 95% chloroform. The lipid mixture was placed on a heat block preheated to 55 °C, and the organic solvent was evaporated under vacuum for 30 min. Dried lipids were resuspended in 500 µL of degassed TBS and incubated at 55 °C with intermittent vortexing for 15 min. The lipid solution was extruded 11 times through a 200-nm filter.

### Buoyant Density Gradient Flotation.

Buoyant density gradient flotation assays were performed as described in (60) with the following modifications. Binding reactions (60 µL) consisted of liposomes (20 µL), purified proteins, and CaCl_2_ (2 mM final) in TBS. The reactions were incubated at room temperature for 15 min, mixed with 60 µL of 60% sucrose in TBS supplemented with 2 mM CaCl_2_ (for a final concentration of 30% sucrose), and sequentially overlaid with 100 µL of 25% sucrose in TBS supplemented with 2 mM CaCl_2_ and 20 µL of TBS supplemented with 2 mM CaCl_2_. The sucrose step gradients were centrifuged in a TLA100 rotor (Beckman) at 100,000 rpm (436,000×*g*) for 10 min at 4 °C. Buoyant liposomes were collected from the top of the gradient, and the bound proteins were analyzed by SDS-PAGE and immunoblotting.

### Statistical Analysis.

Statistical analyses were performed using GraphPad Prism 10. Unpaired two-tailed Student’s *t* tests were used for comparisons between two groups, and one- or two-way ANOVA tests were used for comparisons among multiple groups. The statistical test used for each experiment is detailed in the figure legends.

## Supplementary Material

Appendix 01 (PDF)

Dataset S01 (XLSX)

Dataset S02 (XLSX)

Movie S1. **Laser ablation of U2-OS SRI-mNG cells**. U2-OS cells stably expressing SRI-mNG were subjected to laser ablation. Green: SRI-mNG; Magenta: FM4-64. Image times are relative to the first image taken after laser ablation. Total imaging time: 5 min. Time between acquisitions: 3 s. Scale bars: 5 μm. See corresponding Figure 3A.

Movie S2. **Laser ablation of U2-OS SRI-mNG cells in the presence of extracellular Ca^2+^**. U2-OS cells stably expressing SRI-mNG were subjected to laser ablation in the presence of extracellular Ca^2+^. Green: SRI-mNG; Magenta: FM4-64. Image times are relative to the first image taken after laser ablation. Total imaging time: 5 min. Time between acquisitions: 3 s. Scale bars: 5 μm. See corresponding Figure 3C.

Movie S3. **Laser ablation of U2-OS SRI-mNG cells in the absence of extracellular Ca^2+^**. U2-OS cells stably expressing SRI-mNG were subjected to laser ablation in the absence of extracellular Ca^2+^. Green: SRI-mNG; Magenta: FM4-64. Image times are relative to the first image taken after laser ablation. Total imaging time: 5 min. Time between acquisitions: 3 s. Scale bars: 5 μm. See corresponding Figure 3C.

Movie S4. **Laser ablation of U2-OS SRI-mNG siNT cells**. U2-OS cells stably expressing SRImNG were transfected with a non-targeting siRNA (siNT) and subjected to laser ablation. Green: SRI-mNG; Magenta: FM4-64. Image times are relative to the first image taken after laser ablation. Total imaging time: 5 min. Time between acquisitions: 3 s. Scale bars: 5 μm. See corresponding Figure 5D.

Movie S5. **Laser ablation of U2-OS SRI-mNG siANXA11 cells**. U2-OS cells stably expressing SRI-mNG were transfected with an ANXA11-targeting siRNA (siANXA11) and subjected to laser ablation. Green: SRI-mNG; Magenta: FM4-64. Image times are relative to the first image taken after laser ablation. Total imaging time: 5 min. Time between acquisitions: 3 s. Scale bars: 5 μm. See corresponding Figure 5D.

Movie S6. **Laser ablation of U2-OS ANXA11-mSc siNT cells**. U2-OS cells stably expressing ANXA11-mSc were transfected with a non-targeting siRNA (siNT) and subjected to laser ablation. Green: ANXA11-mSc; Magenta: FM4-64. Image times are relative to the first image taken after laser ablation. Total imaging time: 5 min. Time between acquisitions: 3 s. Scale bars: 5 μm. See corresponding Figure 5F.

Movie S7. **Laser ablation of U2-OS ANXA11-mSc siSRI cells**. U2-OS cells stably expressing ANXA11-mSc were transfected with a sorcin-targeting siRNA (siSRI) and subjected to laser ablation. Green: ANXA11-mSc; Magenta: FM4-64. Image times are relative to the first image taken after laser ablation. Total imaging time: 5 min. Time between acquisitions: 3 s. Scale bars: 5 μm. See corresponding Figure 5F.

Movie S8. **Laser ablation of U2-OS ALIX-mNG siNT cells**. U2-OS cells stably expressing ALIXmNG were transfected with a non-targeting siRNA (siNT) and subjected to laser ablation. Green: ALIX-mNG; Magenta: FM4-64. Image times are relative to the first image taken after laser ablation. Total imaging time: 5 min. Time between acquisitions: 3 s. Scale bars: 5 μm. See corresponding Figure 6A.

Movie S9. **Laser ablation of U2-OS ALIX-mNG siSRI cells**. U2-OS cells stably expressing ALIXmNG were transfected with a sorcin-targeting siRNA (siSRI) and subjected to laser ablation. Green: ALIX-mNG; Magenta: FM4-64. Image times are relative to the first image taken after laser ablation. Total imaging time: 5 min. Time between acquisitions: 3 s. Scale bars: 5 μm. See corresponding Figure 6A.

Movie S10. **Laser ablation of U2-OS ALIX-mNG siANXA11 cells**. U2-OS cells stably expressing ALIX-mNG were transfected with an ANXA11-targeting siRNA (siANXA11) and subjected to laser ablation. Green: ALIX-mNG; Magenta: FM4-64. Image times are relative to the first image taken after laser ablation. Total imaging time: 5 min. Time between acquisitions: 3 s. Scale bars: 5 μm. See corresponding Figure 6A.

Movie S11. **Laser ablation of U2-OS CHMP4B-mNG siNT cells**. U2-OS cells stably expressing CHMP4B-mNG were transfected with a non-targeting siRNA (siNT) and subjected to laser ablation. Green: CHMP4B-mNG; Magenta: FM4-64. Image times are relative to the first image taken after laser ablation. Total imaging time: 5 min. Time between acquisitions: 3 s. Scale bars: 5 μm. See corresponding Figure 6C.

Movie S12. **Laser ablation of U2-OS CHMP4B-mNG siSRI cells**. U2-OS cells stably expressing CHMP4B-mNG were transfected with a sorcin-targeting siRNA (siSRI) and subjected to laser ablation. Green: CHMP4B-mNG; Magenta: FM4-64. Image times are relative to the first image taken after laser ablation. Total imaging time: 5 min. Time between acquisitions: 3 s. Scale bars: 5 μm. See corresponding Figure 6C.

Movie S13. **Laser ablation of U2-OS CHMP4B-mNG siANXA11 cells**. U2-OS cells stably expressing CHMP4B-mNG were transfected with an ANXA11-targeting siRNA (siANXA11) and subjected to laser ablation. Green: CHMP4B-mNG; Magenta: FM4-64. Image times are relative to the first image taken after laser ablation. Total imaging time: 5 min. Time between acquisitions: 3 s. Scale bars: 5 μm. See corresponding Figure 6C.

## Data Availability

All study data are included in the article and/or supporting information.

## References

[1] P. L. McNeil, R. A. Steinhardt, Plasma membrane disruption: Repair, prevention, adaptation. Annu. Rev. Cell Dev. Biol. **19**, 697–731 (2003). 14570587 10.1146/annurev.cellbio.19.111301.140101

[2] N. W. Andrews, M. Corrotte, Plasma membrane repair. Curr. Biol. **28**, R392–R397 (2018). 29689221 10.1016/j.cub.2017.12.034

[3] A. Horn, J. K. Jaiswal, Cellular mechanisms and signals that coordinate plasma membrane repair. Cell. Mol. Life Sci. **75**, 3751–3770 (2018). 30051163 10.1007/s00018-018-2888-7 PMC6541445

[4] C. Dias, J. Nylandsted, Plasma membrane integrity in health and disease: Significance and therapeutic potential. Cell Discov. **7**, 4 (2021). 33462191 10.1038/s41421-020-00233-2 PMC7813858

[5] M. Terasaki, K. Miyake, P. L. McNeil, Large plasma membrane disruptions are rapidly resealed by Ca^2+^-dependent vesicle–vesicle fusion events. J. Cell Biol. **139**, 63–74 (1997). 9314529 10.1083/jcb.139.1.63 PMC2139822

[6] V. Gerke, S. E. Moss, Annexins: From structure to function. Physiol. Rev. **82**, 331–371 (2002). 11917092 10.1152/physrev.00030.2001

[7] V. Gerke , Annexins—A family of proteins with distinctive tastes for cell signaling and membrane dynamics. Nat. Commun. **15**, 1574 (2024). 38383560 10.1038/s41467-024-45954-0 PMC10882027

[8] A. K. McNeil, U. Rescher, V. Gerke, P. L. McNeil, Requirement for annexin A1 in plasma membrane repair. J. Biol. Chem. **281**, 35202–35207 (2006). 16984915 10.1074/jbc.M606406200

[9] A. Bouter , Annexin-A5 assembled into two-dimensional arrays promotes cell membrane repair. Nat. Commun. **2**, 270 (2011). 21468022 10.1038/ncomms1270 PMC3104517

[10] A. R. Demonbreun , An actin-dependent annexin complex mediates plasma membrane repair in muscle. J. Cell Biol. **213**, 705–718 (2016). 27298325 10.1083/jcb.201512022 PMC4915191

[11] S. N. Koerdt, V. Gerke, Annexin A2 is involved in Ca^2+^-dependent plasma membrane repair in primary human endothelial cells. Biochim Biophys. Acta Mol. Cell Res. **1864**, 1046–1053 (2017). 27956131 10.1016/j.bbamcr.2016.12.007

[12] S. J. Foltz, Y. Y. Cui, H. J. Choo, H. C. Hartzell, ANO5 ensures trafficking of annexins in wounded myofibers. J. Cell Biol. **220**, e202007059 (2021). 33496727 10.1083/jcb.202007059 PMC7844426

[13] K. A. Swaggart , Annexin A6 modifies muscular dystrophy by mediating sarcolemmal repair. Proc. Natl. Acad. Sci. U.S.A. **111**, 6004–6009 (2014). 24717843 10.1073/pnas.1324242111 PMC4000833

[14] A. Defour , Annexin A2 links poor myofiber repair with inflammation and adipogenic replacement of the injured muscle. Hum. Mol. Genet. **26**, 1979–1991 (2017). 28334824 10.1093/hmg/ddx065 PMC6075559

[15] C. Croissant, R. Carmeille, C. Brévart, A. Bouter, Annexins and membrane repair dysfunctions in muscular dystrophies. Int. J. Mol. Sci. **22**, 5276 (2021). 34067866 10.3390/ijms22105276 PMC8155887

[16] A. J. Jimenez , ESCRT machinery is required for plasma membrane repair. Science. **343**, 1247136 (2014). 24482116 10.1126/science.1247136

[17] D. J. Katzmann, M. Babst, S. D. Emr, Ubiquitin-dependent sorting into the multivesicular body pathway requires the function of a conserved endosomal protein sorting complex, ESCRT-I. Cell **106**, 145–155 (2001). 11511343 10.1016/s0092-8674(01)00434-2

[18] J. Schöneberg, I. H. Lee, J. H. Iwasa, J. H. Hurley, Reverse-topology membrane scission by the ESCRT proteins. Nat. Rev. Mol. Cell Biol. **18**, 5–17 (2017). 27703243 10.1038/nrm.2016.121 PMC5198518

[19] M. Vietri, M. Radulovic, H. Stenmark, The many functions of ESCRTs. Nat. Rev. Mol. Cell Biol. **21**, 25–42 (2020). 31705132 10.1038/s41580-019-0177-4

[20] Y. Zhen, M. Radulovic, M. Vietri, H. Stenmark, Sealing holes in cellular membranes. EMBO J. **40**, e106922 (2021). 33644904 10.15252/embj.2020106922 PMC8013788

[21] M. Burigotto, J. G. Carlton, ESCRT-III function in membrane fission and repair. Nat. Rev. Mol. Cell Biol. **27**, 297–315 (2025). 41299081 10.1038/s41580-025-00909-1 PMC7618577

[22] M. L. Skowyra, P. H. Schlesinger, T. V. Naismith, P. I. Hanson, Triggered recruitment of ESCRT machinery promotes endolysosomal repair. Science **360**, eaar5078 (2018). 29622626 10.1126/science.aar5078 PMC6195421

[23] C. Goul , LASER couples damage sensing to ESCRT assembly for lysosome repair. Nature, 10.1038/s41586-026-10604-6 (2026). PMC13442042 42236937

[24] L. L. Scheffer , Mechanism of Ca^2+^-triggered ESCRT assembly and regulation of cell membrane repair. Nat. Commun. **5**, 5646 (2014). 25534348 10.1038/ncomms6646 PMC4333728

[25] S. L. Sønder , Annexin A7 is required for ESCRT III-mediated plasma membrane repair. Sci. Rep. **9**, 6726 (2019). 31040365 10.1038/s41598-019-43143-4 PMC6491720

[26] S. Shukla, K. P. Larsen, C. Ou, K. Rose, J. H. Hurley, In vitro reconstitution of calcium-dependent recruitment of the human ESCRT machinery in lysosomal membrane repair. Proc. Natl. Acad. Sci. U.S.A. **119**, e2205590119 (2022). 35994655 10.1073/pnas.2205590119 PMC9436306

[27] S. Shukla , Mechanism and cellular function of direct membrane binding by the ESCRT and ERES-associated Ca^2+^-sensor ALG-2. Proc. Natl. Acad. Sci. U.S.A. **121**, e2318046121 (2024). 38386713 10.1073/pnas.2318046121 PMC10907313

[28] G. Skibinski , Mutations in the endosomal ESCRTIII-complex subunit CHMP2B in frontotemporal dementia. Nat. Genet. **37**, 806–808 (2005). 16041373 10.1038/ng1609

[29] B. N. Smith , Mutations in the vesicular trafficking protein annexin A11 are associated with amyotrophic lateral sclerosis. Sci. Transl. Med. **9**, eaad9157 (2017). 28469040 10.1126/scitranslmed.aad9157 PMC6599403

[30] M. Johari , Adult-onset dominant muscular dystrophy in Greek families caused by annexin A11. Ann. Clin. Transl. Neurol. **9**, 1660–1667 (2022). 36134701 10.1002/acn3.51665 PMC9539373

[31] A. P. Martins , The novel missense variant D40V causes a young adult presentation of ANXA11-related myopathy. Neuromuscul. Disord. **53**, 105443 (2025). 40730020 10.1016/j.nmd.2025.105443

[32] C. M. Heffner , The ALS- and FTD-associated proteins Annexin A11 and CHMP2B act sequentially in plasma membrane repair. *Dev. Cell* **61**, 1487–1503.e7 (2026). 10.1016/j.devcel.2026.05.014 PMC13374510 42349418

[33] J. M. Replogle , Maximizing CRISPRi efficacy and accessibility with dual-sgRNA libraries and optimal effectors. eLife **11**, e81856 (2022). 36576240 10.7554/eLife.81856 PMC9829409

[34] B. A. Johnstone , Cholesterol-dependent cytolysins: The outstanding questions. IUBMB Life **74**, 1169–1179 (2022). 35836358 10.1002/iub.2661 PMC9712165

[35] J. K. Williams , Calpains orchestrate secretion of annexin-containing microvesicles during membrane repair. J. Cell Biol. **224**, e202408159 (2025). 40377476 10.1083/jcb.202408159 PMC12083247

[36] M. Maki, Y. Kitaura, H. Satoh, S. Ohkouchi, H. Shibata, Structures, functions and molecular evolution of the penta-EF-hand Ca2+-binding proteins. Biochim. Biophys. Acta **1600**, 51–60 (2002). 12445459 10.1016/s1570-9639(02)00444-2

[37] A. M. Van der Bliek, M. B. Meyers, J. L. Biedler, E. Hes, P. Borst, A 22-kd protein (sorcin/V19) encoded by an amplified gene in multidrug-resistant cells, is homologous to the calcium-binding light chain of calpain. EMBO J. **5**, 3201–3208 (1986). 3028774 10.1002/j.1460-2075.1986.tb04630.x PMC1167313

[38] M. B. Meyers , Association of sorcin with the cardiac ryanodine receptor. J. Biol. Chem. **270**, 26411–26418 (1995). 7592856 10.1074/jbc.270.44.26411

[39] A. J. Lokuta, M. B. Meyers, P. R. Sander, G. I. Fishman, H. H. Valdivia, Modulation of cardiac ryanodine receptors by sorcin. J. Biol. Chem. **272**, 25333–25338 (1997). 9312152 10.1074/jbc.272.40.25333

[40] E. F. Farrell, A. Antaramian, A. Rueda, A. M. Gómez, H. H. Valdivia, Sorcin inhibits calcium release and modulates excitation-contraction coupling in the heart. J. Biol. Chem. **278**, 34660–34666 (2003). 12824171 10.1074/jbc.M305931200

[41] T. Matsumoto , Sorcin interacts with sarcoplasmic reticulum Ca^2+^-ATPase and modulates excitation-contraction coupling in the heart. Basic Res. Cardiol. **100**, 250–262 (2005). 15754088 10.1007/s00395-005-0518-7

[42] G. Colotti , Sorcin, a calcium binding protein involved in the multidrug resistance mechanisms in cancer cells. Molecules **19**, 13976–13989 (2014). 25197934 10.3390/molecules190913976 PMC6271628

[43] A. M. Brownawell, C. E. Creutz, Calcium-dependent binding of sorcin to the N terminal domain of synexin (annexin VII). J. Biol. Chem. **272**, 22182–22190 (1997). 9268363 10.1074/jbc.272.35.22182

[44] M. Missotten, A. Nichols, K. Rieger, R. Sadoul, Alix, a novel mouse protein undergoing calcium-dependent interaction with the apoptosis-linked-gene 2 (ALG-2) protein. Cell Death Differ. **6**, 124–129 (1999). 10200558 10.1038/sj.cdd.4400456

[45] M. Maki, Y. Maemoto, Y. Osako, H. Shibata, Evolutionary and physical linkage between calpains and penta-EF-hand Ca^2+^-binding proteins. FEBS J. **279**, 1414–1421 (2012). 22404899 10.1111/j.1742-4658.2012.08560.x

[46] S. Tarabykina, A. L. Møller, I. Durussel, J. Cox, M. W. Berchtold, Two forms of the apoptosis-linked protein ALG-2 with different Ca^2+^ affinities and target recognition. J. Biol. Chem. **275**, 10514–10518 (2000). 10744743 10.1074/jbc.275.14.10514

[47] H. Satoh, Y. Nakano, H. Shibata, M. Maki, The penta-EF-hand domain of ALG-2 interacts with amino-terminal domains of both annexin VII and annexin XI in a Ca^2+^-dependent manner. Biochim. Biophys. Acta **1600** (1–2), 61–67 (2002). 12445460 10.1016/s1570-9639(02)00445-4

[48] J. Van der Zee , CHMP2B C-truncating mutations in frontotemporal lobar degeneration are associated with an aberrant endosomal phenotype in vitro. Hum. Mol. Genet. **17**, 313–322 (2008). 17956895 10.1093/hmg/ddm309

[49] C. Rodger , De Novo VPS4A mutations cause multisystem disease with abnormal neurodevelopment. Am. J. Hum. Genet. **107**, 1129–1148 (2020). 33186545 10.1016/j.ajhg.2020.10.012 PMC7820634

[50] A. N. Coyne , Nuclear accumulation of CHMP7 initiates nuclear pore complex injury and subsequent TDP-43 dysfunction in sporadic and familial ALS. Sci. Transl. Med., **13**, eabe1923 (2021). 34321318 10.1126/scitranslmed.abe1923 PMC9022198

[51] A. N. Coyne, J. D. Rothstein, The ESCRT-III protein VPS4, but not CHMP4B or CHMP2B, is pathologically increased in familial and sporadic ALS neuronal nuclei. Acta Neuropathol. Commun. **9**, 127 (2021). 34281622 10.1186/s40478-021-01228-0 PMC8287756

[52] O. Keeley, A. N. Coyne, Nuclear and degradative functions of the ESCRT-III pathway: Implications for neurodegenerative disease. Nucleus **15**, 2349085 (2024). 38700207 10.1080/19491034.2024.2349085 PMC11073439

[53] H. G. Göttlinger, T. Dorfman, J. G. Sodroski, W. A. Haseltine, Effect of mutations affecting the p6 gag protein on human immunodeficiency virus particle release. Proc. Natl. Acad. Sci. U.S.A. **88**, 3195–3199 (1991). 2014240 10.1073/pnas.88.8.3195 PMC51412

[54] J. E. Garrus , Tsg101 and the vacuolar protein sorting pathway are essential for HIV-1 budding. Cell **107**, 55–65 (2001). 11595185 10.1016/s0092-8674(01)00506-2

[55] B. Strack, A. Calistri, S. Craig, E. Popova, H. G. Göttlinger, AIP1/ALIX is a binding partner for HIV-1 p6 and EIAV p9 functioning in virus budding. Cell **114**, 689–699 (2003). 14505569 10.1016/s0092-8674(03)00653-6

[56] R. D. Fisher , Structural and biochemical studies of ALIX/AIP1 and its role in retrovirus budding. Cell **128**, 841–852 (2007). 17350572 10.1016/j.cell.2007.01.035

[57] J. K. Williams, J. M. Ngo, I. M. Lehman, R. Schekman, Annexin A6 mediates calcium-dependent exosome secretion during plasma membrane repair. eLife **12**, e86556 (2023). 37204294 10.7554/eLife.86556 PMC10241516

[58] J. M. Ngo , Extracellular vesicles and cellular homeostasis. Annu. Rev. Biochem. **94**, 587–609 (2025). 40101210 10.1146/annurev-biochem-100924-012717

[59] J. M. Ngo, J. K. Williams, M. M. Temoche-Diaz, A. Murugupandiyan, R. Schekman, p62 sorts Lupus La and selected microRNAs into breast cancer-derived exosomes. J. Cell Biol. **225**, e202503087 (2026). 41474625 10.1083/jcb.202503087 PMC12755392

[60] P. Marchando , Polarized anionic phospholipids and exocytosis are implicated in the polarized recruitment of budding yeast AP180, an endocytic initiator. Mol. Biol Cell **37**, br17 (2026). 41949887 10.1091/mbc.E24-10-0446 PMC13200705

