# Supplementary material for: Sorcin couples Annexin A11 recruitment to ESCRT-III assembly for plasma membrane repair
Source: Proc Natl Acad Sci U S A. 2026 Aug 10;123(33):e2610420123. doi: 10.1073/pnas.2610420123 (PMC13486606; doi:10.1073/pnas.2610420123)
Supplement: Supplementary file 1 [file pnas.2610420123.sapp.pdf]

SUPPORTING INFORMATION

Movies

Movie 1. Laser ablation of U2-OS SRI-mNG cells. U2-OS cells stably expressing SRI-mNG were subjected to laser ablation. Green: SRI-mNG; Magenta: FM4-64. Image times are relative to the first image taken after laser ablation. Total imaging time: 5 min. Time between acquisitions: 3 s. Scale bars: 5 µm. See corresponding Figure 3A.

Movie 2. Laser ablation of U2-OS SRI-mNG cells in the presence of extracellular Ca^{2+}. U2-OS cells stably expressing SRI-mNG were subjected to laser ablation in the presence of extracellular Ca^{2+}. Green: SRI-mNG; Magenta: FM4-64. Image times are relative to the first image taken after laser ablation. Total imaging time: 5 min. Time between acquisitions: 3 s. Scale bars: 5 µm. See corresponding Figure 3C.

Movie 3. Laser ablation of U2-OS SRI-mNG cells in the absence of extracellular Ca^{2+}. U2-OS cells stably expressing SRI-mNG were subjected to laser ablation in the absence of extracellular Ca^{2+}. Green: SRI-mNG; Magenta: FM4-64. Image times are relative to the first image taken after laser ablation. Total imaging time: 5 min. Time between acquisitions: 3 s. Scale bars: 5 µm. See corresponding Figure 3C.

Movie 4. Laser ablation of U2-OS SRI-mNG siNT cells. U2-OS cells stably expressing SRI-mNG were transfected with a non-targeting siRNA (siNT) and subjected to laser ablation. Green: SRI-mNG; Magenta: FM4-64. Image times are relative to the first image taken after laser ablation. Total imaging time: 5 min. Time between acquisitions: 3 s. Scale bars: 5 µm. See corresponding Figure 5D.

Movie 5. Laser ablation of U2-OS SRI-mNG siANXA11 cells. U2-OS cells stably expressing SRI-mNG were transfected with an ANXA11-targeting siRNA (siANXA11) and subjected to laser ablation. Green: SRI-mNG; Magenta: FM4-64. Image times are relative to the first image taken after laser ablation. Total imaging time: 5 min. Time between acquisitions: 3 s. Scale bars: 5 µm. See corresponding Figure 5D.

Movie 6. Laser ablation of U2-OS ANXA11-mSc siNT cells. U2-OS cells stably expressing ANXA11-mSc were transfected with a non-targeting siRNA (siNT) and subjected to laser ablation. Green: ANXA11-mSc; Magenta: FM4-64. Image times are relative to the first image taken after laser ablation. Total imaging time: 5 min. Time between acquisitions: 3 s. Scale bars: 5 µm. See corresponding Figure 5F.

Movie 7. Laser ablation of U2-OS ANXA11-mSc siSRI cells. U2-OS cells stably expressing ANXA11-mSc were transfected with a sorcin-targeting siRNA (siSRI) and subjected to laser ablation. Green: ANXA11-mSc; Magenta: FM4-64. Image times are relative to the first image taken after laser ablation. Total imaging time: 5 min. Time between acquisitions: 3 s. Scale bars: 5 µm. See corresponding Figure 5F.

Movie 8. Laser ablation of U2-OS ALIX-mNG siNT cells. U2-OS cells stably expressing ALIX-mNG were transfected with a non-targeting siRNA (siNT) and subjected to laser ablation. Green: ALIX-mNG; Magenta: FM4-64. Image times are relative to the first image taken after laser ablation. Total imaging time: 5 min. Time between acquisitions: 3 s. Scale bars: 5 μm. See corresponding Figure 6A.

Movie 9. Laser ablation of U2-OS ALIX-mNG siSRI cells. U2-OS cells stably expressing ALIX-mNG were transfected with a sorcin-targeting siRNA (siSRI) and subjected to laser ablation. Green: ALIX-mNG; Magenta: FM4-64. Image times are relative to the first image taken after laser ablation. Total imaging time: 5 min. Time between acquisitions: 3 s. Scale bars: 5 μm. See corresponding Figure 6A.

Movie 10. Laser ablation of U2-OS ALIX-mNG siANXA11 cells. U2-OS cells stably expressing ALIX-mNG were transfected with an ANXA11-targeting siRNA (siANXA11) and subjected to laser ablation. Green: ALIX-mNG; Magenta: FM4-64. Image times are relative to the first image taken after laser ablation. Total imaging time: 5 min. Time between acquisitions: 3 s. Scale bars: 5 μm. See corresponding Figure 6A.

Movie 11. Laser ablation of U2-OS CHMP4B-mNG siNT cells. U2-OS cells stably expressing CHMP4B-mNG were transfected with a non-targeting siRNA (siNT) and subjected to laser ablation. Green: CHMP4B-mNG; Magenta: FM4-64. Image times are relative to the first image taken after laser ablation. Total imaging time: 5 min. Time between acquisitions: 3 s. Scale bars: 5 μm. See corresponding Figure 6C.

Movie 12. Laser ablation of U2-OS CHMP4B-mNG siSRI cells. U2-OS cells stably expressing CHMP4B-mNG were transfected with a sorcin-targeting siRNA (siSRI) and subjected to laser ablation. Green: CHMP4B-mNG; Magenta: FM4-64. Image times are relative to the first image taken after laser ablation. Total imaging time: 5 min. Time between acquisitions: 3 s. Scale bars: 5 μm. See corresponding Figure 6C.

Movie 13. Laser ablation of U2-OS CHMP4B-mNG siANXA11 cells. U2-OS cells stably expressing CHMP4B-mNG were transfected with an ANXA11-targeting siRNA (siANXA11) and subjected to laser ablation. Green: CHMP4B-mNG; Magenta: FM4-64. Image times are relative to the first image taken after laser ablation. Total imaging time: 5 min. Time between acquisitions: 3 s. Scale bars: 5 μm. See corresponding Figure 6C.

Tables

Table 1. Quantitative proteomics of inside-out PM vesicles in the absence or presence of Ca^{2+}.

Table 2. Genome-wide CRISPR interference screens for cell survival after PM damage.
